# SIK2 inhibition enhances PARP inhibitor activity synergistically in ovarian and triple-negative breast cancers

**DOI:** 10.1172/JCI146471

**Published:** 2022-06-01

**Authors:** Zhen Lu, Weiqun Mao, Hailing Yang, Janice M. Santiago-O’Farrill, Philip J. Rask, Jayanta Mondal, Hu Chen, Cristina Ivan, Xiuping Liu, Chang-Gong Liu, Yuanxin Xi, Kenta Masuda, Eli M. Carrami, Meng Chen, Yitao Tang, Lan Pang, David S. Lakomy, George A. Calin, Han Liang, Ahmed A. Ahmed, Hariprasad Vankayalapati, Robert C. Bast

**Affiliations:** 1 Department of Experimental Therapeutics,; 2 Department of Bioinformatics & Computational Biology, and; 3 Department of Translational Molecular Pathology, University of Texas MD Anderson Cancer Center, Houston, Texas, USA.; 4 The University of Texas MD Anderson UTHealth Graduate School of Biomedical Sciences, Houston, Texas, USA.; 5 Ovarian Cancer Cell Laboratory, Weatherall Institute of Molecular Medicine, University of Oxford, Headington, Oxford, United Kingdom.; 6 Department of Systems Biology, University of Texas MD Anderson Cancer Center, Houston, Texas, USA.; 7 Nuffield Department of Women’s & Reproductive Health, University of Oxford, Women’s Centre, John Radcliffe Hospital, Oxford, United Kingdom.; 8 Oxford NIHR Biomedical Research Centre, Oxford, United Kingdom.; 9 Arrien Pharmaceuticals, Salt Lake City, Utah, USA.

**Keywords:** Cell Biology, Therapeutics, Apoptosis, Cancer, DNA repair

## Abstract

Poly(ADP-ribose) polymerase inhibitors (PARP inhibitors) have had an increasing role in the treatment of ovarian and breast cancers. PARP inhibitors are selectively active in cells with homologous recombination DNA repair deficiency caused by mutations in *BRCA1/2* and other DNA repair pathway genes. Cancers with homologous recombination DNA repair proficiency respond poorly to PARP inhibitors. Cancers that initially respond to PARP inhibitors eventually develop drug resistance. We have identified salt-inducible kinase 2 (SIK2) inhibitors, ARN3236 and ARN3261, which decreased DNA double-strand break (DSB) repair functions and produced synthetic lethality with multiple PARP inhibitors in both homologous recombination DNA repair deficiency and proficiency cancer cells. SIK2 is required for centrosome splitting and PI3K activation and regulates cancer cell proliferation, metastasis, and sensitivity to chemotherapy. Here, we showed that SIK2 inhibitors sensitized ovarian and triple-negative breast cancer (TNBC) cells and xenografts to PARP inhibitors. SIK2 inhibitors decreased PARP enzyme activity and phosphorylation of class-IIa histone deacetylases (HDAC4/5/7). Furthermore, SIK2 inhibitors abolished class-IIa HDAC4/5/7–associated transcriptional activity of myocyte enhancer factor-2D (MEF2D), decreasing MEF2D binding to regulatory regions with high chromatin accessibility in *FANCD2*, *EXO1*, and *XRCC4* genes, resulting in repression of their functions in the DNA DSB repair pathway. The combination of PARP inhibitors and SIK2 inhibitors provides a therapeutic strategy to enhance PARP inhibitor sensitivity for ovarian cancer and TNBC.

## Graphical abstract

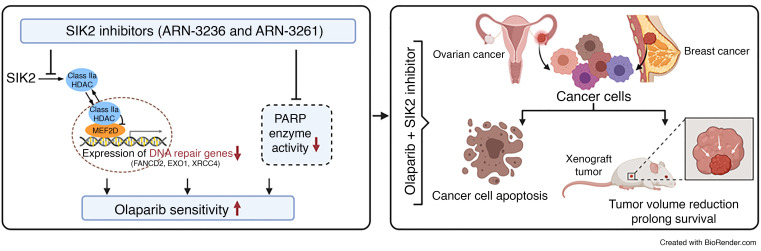

## Introduction

Recent studies indicate that DNA damage, aberrations in the DNA damage response, and defects in DNA repair machinery play a major role in ovarian cancer and triple-negative breast cancer (TNBC, refs. 1, 2). DNA double-strand breaks (DSBs) are considered one of the most cytotoxic forms of DNA damage that can lead to mutation and trigger permanent growth arrest or cell death (3). The 2 main DSB repair pathways are nonhomologous end-joining (NHEJ) and homologous recombination (4–6). NHEJ is a rapid, high-capacity pathway that joins 2 DNA ends using the ligase IV/XRCC4 (x-ray repair cross complementing 4) complex that recognizes DSBs (7). NHEJ can accommodate very limited base pairing between the 2 processed DNA ends, thereby potentially forming repair joints with as few as 4 bp of microhomology (4). By contrast, homologous recombination requires extensive sequence homology between the broken DNA and a donor DNA molecule. The end resection regulated by exonuclease 1 (EXO1) at DSBs and the DNA synthesis using intact homologous DNA sequence as templates are the key steps in the homologous recombination DNA repair process (8, 9). The Fanconi anemia (FA) pathway is closely linked to homologous recombination DNA repair through its functional interaction with BRCA1/2 (10). FA-group D2 (FANCD2) protein promotes homologous recombination DNA repair and prevents DNA DSB formation and chromosomal aberrations in DNA-damaged cells (9). Most DNA repair pathways are complex, involving many proteins working in discrete consecutive steps. Therefore, the efficiency of DNA repair requires transcription factors controlling and maintaining the expression of DNA repair genes. DNA DSB repair is a critical prerequisite for cancer cell survival, and its dysregulation in cancer cells could provide important therapeutic targets.

Class-IIa histone deacetylases (HDACs) are involved in the regulation of multiple cellular responses, including DNA repair. HDACs generally regulate particular genetic programs by influencing the landscape of genes expressed in a specific context. Class-IIa HDACs do not bind directly to DNA, but rather interact with a limited number of transcription factors, such as myocyte enhancer factor-2 (MEF2), which are recruited to specific genomic regions in a sequence-dependent manner (11). MEF2 is a MADS box transcription factor originally discovered as a regulator of cardiogenesis and myogenesis (12). MEF2 influences the expression of numerous genes, individually and cooperatively with other transcription factors, including genes involved in DNA repair in normal cells (12). MEF2 can also operate as a transcriptional repressor when complexed with class-IIa HDACs (13). However, the link between the repressor function of the MEF2/class-IIa HDAC axis and expression of DNA repair genes in cancers is not well established.

Salt-inducible kinase 2 (SIK2) is an AMPK-related protein kinase that is required for ovarian cancer cell proliferation and metastasis (14). The kinase phosphorylates multiple substrates, including centrosomal Nek2-associated protein 1, to trigger centrosome splitting and the regulatory subunit of PI3K to enhance the PI3K pathway’s activity (15, 16). SIK2 also phosphorylates class-IIa HDACs and controls their nuclear-cytoplasm shuttling, thus influencing the activity and nuclear localization of class-IIa HDACs (17). SIK2 is overexpressed in a fraction of patients with high-grade serous ovarian carcinoma (HGSOC) and correlates with a poor prognosis (15). In collaboration with Arrien Pharmaceuticals, we have developed orally administered low-MW drugs (ARN3236 and ARN3261) that inhibit SIK2 at nanomolar concentrations, inhibit growth of ovarian cancer cell lines with an IC_50_ of 0.8 to 2.6 μM, and inhibit growth of ovarian cancer xenografts, enhancing sensitivity to paclitaxel (18) and carboplatin (19).

Approximately half of HGSOCs and TNBCs exhibit aberrations in the homologous recombination and other DNA DSB repair pathways (20, 21). HGSOC and TNBC with mutations of *BRCA1* or *BRCA2* are highly sensitive to poly(ADP-ribose) polymerase inhibitors (PARP inhibitors). A fraction of HGSOCs and TNBCs without *BRCA1/2* mutations have homologous recombination DNA repair deficiency and are also susceptible to PARP inhibitors (21, 22). Three PARP inhibitors (olaparib, rucaparib, and niraparib) have been approved by the FDA for use in ovarian cancer (23, 24), and 2 PARP inhibitors (olaparib and talazoparib) have been approved for use in breast cancer (23). Despite promising clinical results for PARP inhibitors as single agents, particularly in cancers with *BRCA1/2* mutations, the myelotoxicity and the high prevalence of acquired resistance remain challenges to more effective treatment. Combination therapies are of considerable interest for minimizing PARP inhibitor concentration and enhancing efficacy. We have found that SIK2 inhibitors enhance response to PARP inhibitors in ovarian cancer and TNBC cell lines and xenografts, independent of *BRCA* mutation status.

## Results

### SIK2 inhibition sensitizes ovarian and breast cancer cells by enhancing olaparib-mediated inhibition of PARP enzyme activity.

**Figure 1:**
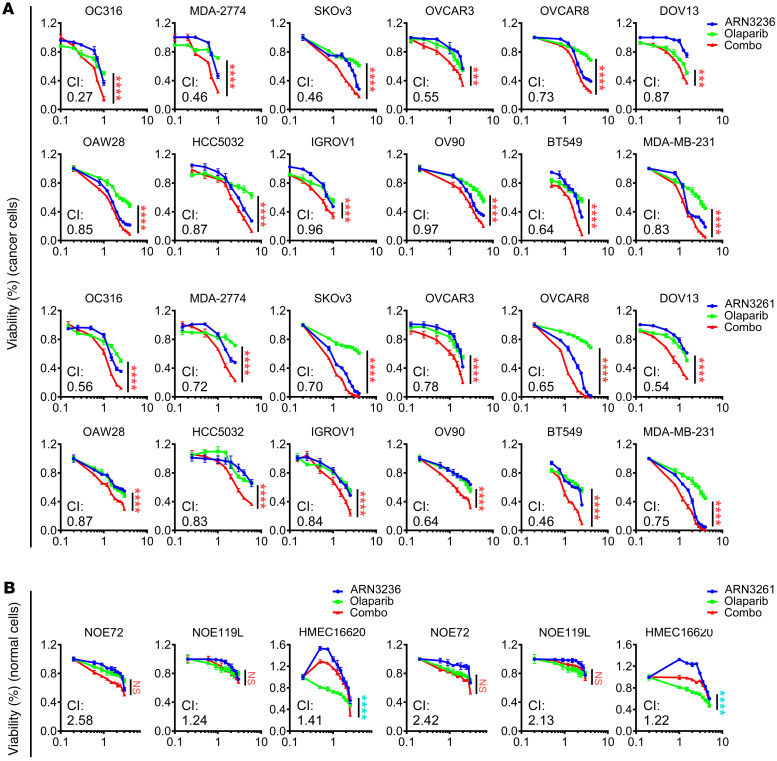
SIK2 inhibitors enhance olaparib sensitivity in ovarian cancer and breast cancer cells. (**A** and **B**) Dose-response curves for ARN3236 or ARN3261 (blue), olaparib (green), or ARN3236 or ARN3261 combined with olaparib (red) for 96 hours in 12 cancer cell lines (**A**) and 3 nonmalignant cell lines (**B**). The IC_50_ of inhibitors and concentration ratio of SIK2 inhibitors to olaparib used in each cell line are listed in Supplemental Table 2. The statistical significance between olaparib alone and SIK2 inhibitor combined with olaparib was calculated with 2-way ANOVA and Tukey’s multiple-comparison test. NS, *P* > 0.05; ***P < 0.001; ****P < 0.0001 (red stars indicate SIK2 inhibitor + olaparib enhancing the effect of olaparib alone; blue stars indicate SIK2 inhibitor + olaparib inhibiting olaparib’s effect). A combination index (CI) at ED_90_ (determination of the 90% effective dose) was calculated using CalcuSyn software. Experiments were repeated 3 times. Representative data were from 1 independent experiment with 4 technical repeats.

**Figure 2:**
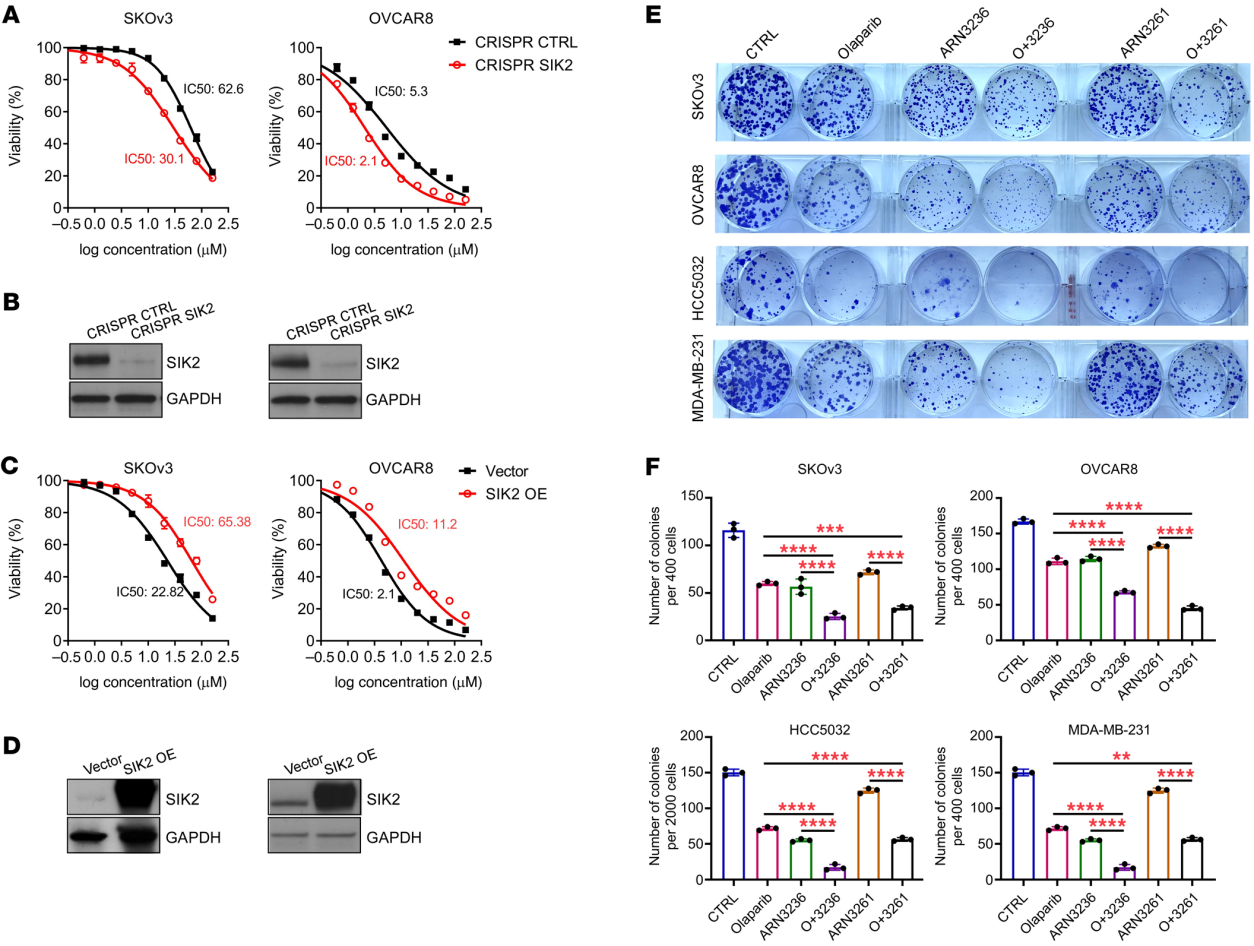
SIK2 expression promotes tumor cell growth, and inhibition of SIK2 enhances olaparib sensitivity in ovarian cancer and breast cancer cells. (**A**–**D**) Dose-response curves of olaparib in paired cancer cell lines with or without KO of SIK2 (**A** and **B**) and with or without stable transfection of SIK2 (**C** and **D**). The IC_50_ for olaparib was calculated using GraphPad Prism 8. Representative data from 1 experiment with 4 replicates are presented. Experiments were repeated 3 times with similar results. Western blot analysis confirmed either SIK2 KO (**B**) or overexpression (**D**). (**E** and **F**) Representative images of clonogenic assays (**E**) and quantification of colonies (**F**) in 4 cancer cell lines are presented. SKOv3, OVCAR8, HCC5032, and MDA-MB-231 cells were treated with olaparib, ARN3236, ARN3261, or olaparib plus ARN3236 at concentrations indicated in Supplemental Figure 1B for 10–22 days. The columns indicate the mean of colonies and the bars indicate the SD. The statistical significance was calculated with 1-way ANOVA and Tukey’s multiple-comparison test (**P < 0.01; ***P < 0.001; ****P < 0.0001). The data were obtained from 1 independent experiment with 3 technical repeats, and experiments were repeated at least 3 times.

**Figure 3:**
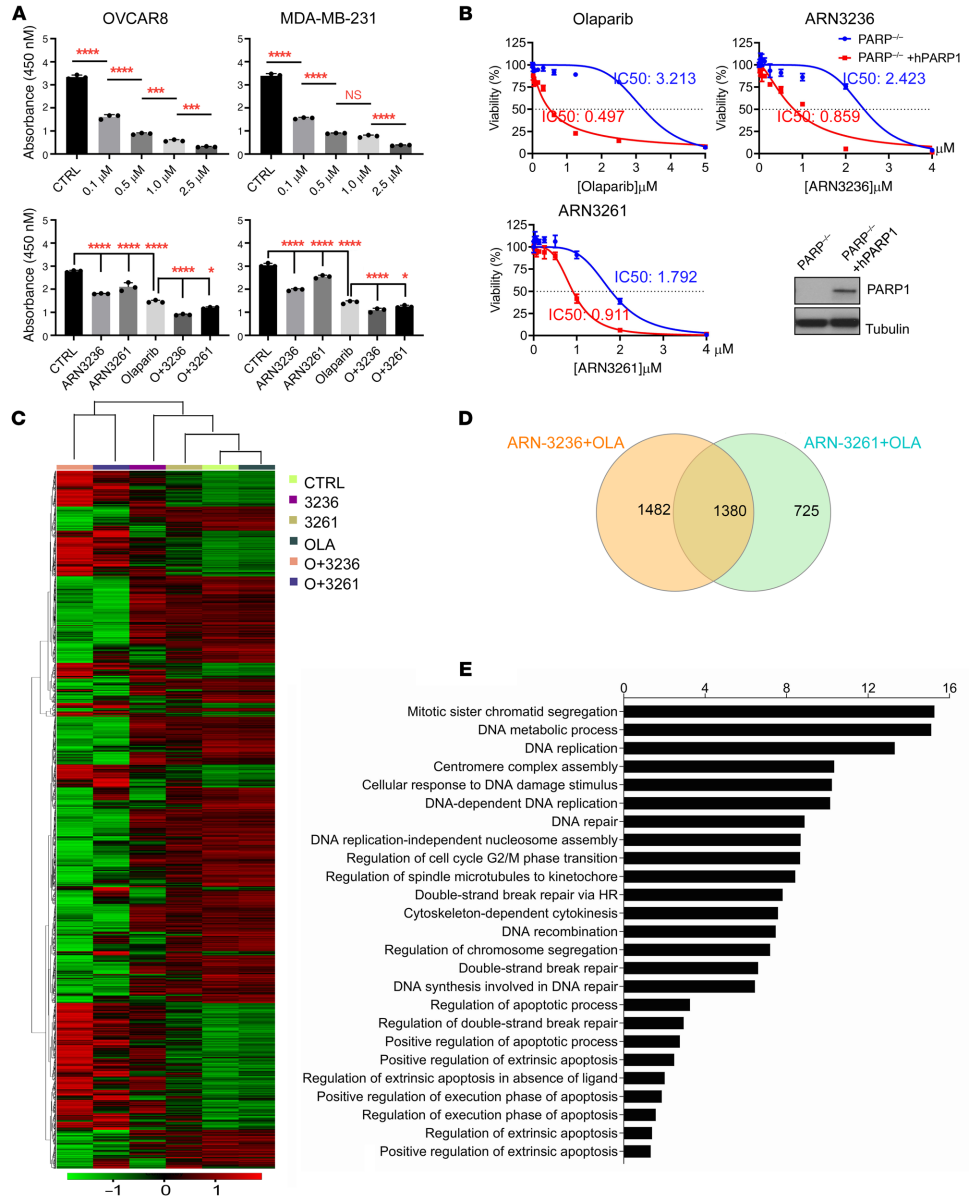
Combined effect of SIK2 inhibitor and olaparib on PARP-1 enzyme activity and DNA DSB repair pathways. (**A**) Dose-response curves for olaparib and combined effect of SIK2 inhibitors with olaparib on PARP-1 enzyme activity. OVCAR8 and MDA-MB-231 cells were treated with SIK2 inhibitors, olaparib alone, or the combination for 26 hours. The concentrations of ARN3236, ARN3261, and olaparib were 6 μM, 4 μM, and 0.05 μM, respectively (also see Supplemental Figure 2C). The columns indicate the mean activity and the bars indicate the SD. The statistical significance was calculated with 1-way ANOVA and Tukey’s multiple-comparison test (*P < 0.05; **P < 0.01; ***P < 0.001; ****P < 0.0001). (**B**) Dose-response curves of ARN3236, ARN3261, and olaparib in DT40 PARP-1^–/–^ cells with and without knockin of human PARP-1 (hPARP). The IC_50_ indicated on the curves was calculated using GraphPad Prism 8. The expression of exogenous hPARP in DT40 PARP-1^–/–^ was measured by Western blotting. For both **A** and **B**, the representative data were from 1 experiment with 3 replicates. Experiments were repeated 3 times with similar results. (**C**) The heatmap presentation of unsupervised hierarchical clustering of gene expression. The heatmap includes 3587 transcripts (upregulated or downregulated by ≥2-fold) treated with ARN3236, ARN3261, olaparib, ARN3236 plus olaparib, and ARN3261 plus olaparib. The heatmap illustrates changes that are color coded with red corresponding to upregulation and green to downregulation. (**D**) The Venn representation. Venn diagram analysis represented the number of genes (upregulated or downregulated by ≥2-fold) that were overlapped by the treatment of ARN3236 plus olaparib (yellow) or ARN3261 plus olaparib (green). (**E**) GO analysis of 1380 differentially expressed genes shared by ARN3236 plus olaparib or ARN3261 plus olaparib treatments. The bar plot shows the log_10_ *P* value of the biological process GO terms obtained with differentially expressed genes at *P* < 0.01.

To determine whether inhibition of SIK2 kinase activity can sensitize cancer cells to PARP inhibitors, we examined the effect of combining a SIK2 kinase inhibitor (ARN3236 or ARN3261) with the PARP inhibitor olaparib on cell growth in 10 ovarian cancer and 8 TNBC cell lines, as well as in normal ovarian and breast cell lines. Olaparib-induced growth inhibition (green line) was significantly enhanced by combined treatment (red line) with either ARN3236 or ARN3261 in all 18 cancer cell lines tested (Figure 1A and Supplemental Figure 1A; supplemental material available online with this article; https://doi.org/10.1172/JCI146471DS1), but not in nontumorigenic NOE72 and NOE119L (normal ovarian epithelial cells) and HMEC16620 (human mammary epithelial cells) (Figure 1B). Importantly, comparing 4 *BRCA1* mutated (HCC1739, MDA-MB-436, Sum1315MO2, and Sum149PT) and 4 *BRCA1* wild-type (BT549, MDA-MB-231, MDA-MB-436, and CAL15) TNBC cell lines, ARN3261 significantly inhibited tumor cell growth in all 8 cell lines tested regardless of *BRCA1* mutation status (Figure 1A and Supplemental Figure 1A). Moreover, all 18 breast and ovarian cancer cell lines exhibited synergistic growth inhibition with a combination of ARN3236 or ARN3261 with olaparib (combination index < 1 using the CalcuSyn model) (Figure 1A and Supplemental Figure 1A), when compared with nontumorigenic cells that did not exhibit synergistic growth inhibition (Figure 1B). To exclude potential off-target effects of SIK2 inhibitors, we knocked out endogenous SIK2 by CRISPR/Cas9 and established stable ectopic expression of SIK2 in SKOv3 and OVCAR8 ovarian cancer cells. Knockout of SIK2 sensitized cancer cells to olaparib judged by lower IC_50_ for olaparib in SIK2-deficient cells compared with control cells (Figure 2, A and B). By contrast, stable ectopic expression of SIK2 in SKOv3 and OVCAR8 cell lines desensitized cancer cells to olaparib, evidenced by an increased IC_50_ (Figure 2, C and D). Clonogenic assays were performed using 3 ovarian cancer cell lines and 1 TNBC cell line. Combination treatment with a SIK2 inhibitor and olaparib significantly decreased the number and size of colonies when compared with either the SIK2 inhibitor or olaparib alone (Figure 2, E and F, and Supplemental Figure 1B). Further, synergistic activity of SIK2 inhibition with PARP inhibition was evaluated with 3 structurally distinct PARP inhibitors (rucaparib, niraparib, and talazoparib) that have different PARP trapping potential (25, 26). Although clinical PARP inhibitors can be ranked by their ability to trap PARP (from the most to the least potent: talazoparib >> niraparib > olaparib = rucaparib) (25, 27), SIK2 inhibitors synergized with PARP inhibitors with high (talazoparib) and low PARP trapping activity (olaparib), exhibiting similar combination indices (Supplemental Figure 1C). PARP binding in the chromatin fraction (indicative of PARP trapping) remained unchanged after treatment with SIK2 inhibitors, suggesting that SIK2 inhibitor–mediated enhancement of PARP inhibition was independent of PARP trapping activity (Supplemental Figure 2A). Measurement of PARP enzyme activity did, however, indicate that treatment with SIK2 inhibitors further decreased olaparib-induced suppression of PARP enzyme activity (Figure 3A) in cancer cells with detectable PARP protein levels (Supplemental Figure 2, B and C), consistent with the possibility that inhibition of PARP enzyme activity underlies, at least in part, the synergistic effect of SIK2 and PARP inhibition. To test this possibility, when we treated DT40 *PARP1*^–/–^ cells that lack PARP enzyme activity (avian cells lack *PARP2*) with SIK2 inhibitors or olaparib, DT40 *PARP1*^–/–^ cells were resistant to olaparib or SIK2 inhibitors (Figure 3B). This was consistent with a requirement for the presence of PARP protein and PARP enzyme activity to observe a synergistic interaction between SIK2 inhibitors and olaparib.

### ARN3236 and ARN3261 perturb transcription of DNA repair and apoptosis genes.

While treatment with the SIK2 inhibitor enhanced olaparib-mediated inhibition of PARP enzyme activity, we asked whether SIK2 inhibitor in combination with PARP inhibitors might alter expression of other key functional components of the DNA DSB repair pathways, contributing to the synergy observed between SIK2 and PARP inhibition. RNA-Seq was performed on SKOv3 ovarian cancer cells treated with vehicle, SIK2 inhibitor, PARP inhibitors, or the combination. The numbers of transcripts upregulated or downregulated 2-fold or more after treatment with ARN3236, ARN3261, olaparib, ARN3236 plus olaparib, or ARN3261 plus olaparib were 1308, 366, 3, 2862, and 2105, respectively. Based on a heatmap with unsupervised hierarchical clustering of 3587 transcripts altered by both ARN3236 plus olaparib and ARN3261 plus olaparib treatments (Figure 3C), olaparib-treated and control groups shared relatively similar transcriptomes, whereas both SIK2 inhibitor and olaparib combination treatment groups clustered together. Combined treatment showed the most significant alteration of transcripts compared with single agents alone, and SIK2 inhibition significantly induced transcriptional repression (Figure 3C). Using a Venn analysis, 1380 differentially expressed transcripts were shared by both SIK2 inhibitors and the olaparib combination treatment groups (Figure 3D). Gene Ontology (GO) biological process enrichment analysis of 1380 differentially expressed genes identified multiple aspects of regulation involving mitosis, DNA damage checkpoint, cell cycle, DNA repair, and apoptosis (Figure 3E), suggesting that SIK2 inhibition may enhance olaparib sensitivity by regulating DNA repair and apoptosis.

### ARN3236 and ARN3261 enhance olaparib-induced DNA DSB and apoptosis.

**Figure 4:**
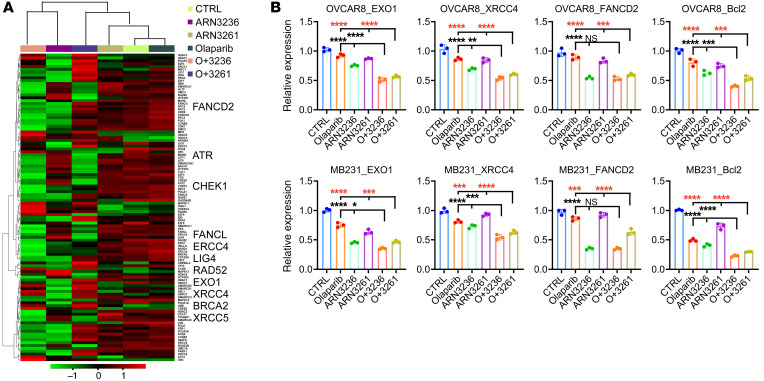
ARN3236 and ARN3261 enhance olaparib-induced DNA DSBs. (**A**) The heatmap representation of unsupervised hierarchical clustering of differentially expressed genes associated with DNA repair. The heatmap illustrates changes that are color coded with red corresponding to upregulation and green to downregulation. (**B**) Analysis of DNA repair and apoptosis genes using RT-PCR. Cells were treated with a single agent or the combination for 72 hours. The concentrations of ARN3236, ARN3261, and olaparib were 4 μM (2 times), 4 μM (3 times), and 15 μM (2 times), respectively. Representative data are from 1 experiment with 3 technical repeats per treatment. Experiments were repeated 3 times. One-way ANOVA and Tukey’s multiple-comparison test were performed (*P < 0.05; **P < 0.01; ***P < 0.001; ****P < 0.0001).

Detailed analysis of the expression of transcripts participating in regulation of DNA repair and apoptosis further demonstrated that SIK2 inhibition enhanced PARP inhibition–mediated increase in DNA DSBs (Figure 4A) and apoptosis (Supplemental Figure 3A). To verify the RNA-Seq results, 9 genes involved in regulation of DNA repair and apoptosis (*BRCA2*, *EXO1*, *FANCD2*, *LIG4*, *XRCC4*, *BAX*, *BCL2*, *CASP7*, and *TRADD*) were selected and analyzed with quantitative reverse-transcription PCR (RT-qPCR) using OVCAR8 ovarian cancer and MDA-MB-231 breast cancer cells. Treatment with ARN3236 or ARN3261 combined with olaparib (ARN3236 plus olaparib or ARN3261 plus olaparib) significantly decreased the expression of *EXO1*, *XRCC4*, *FANCD2*, *BRCA2*, *LIG4*, *CASP7*, and *BCL2* and increased expression of *BAX* compared with olaparib treatment alone in both cell lines tested (Figure 4B and Supplemental Figure 3B). Similar results were also observed in the cells treated with ARN3261 in combination with olaparib (Figure 4B and Supplemental Figure 3B). These data are consistent with data obtained by RNA-Seq analysis.

**Figure 5:**
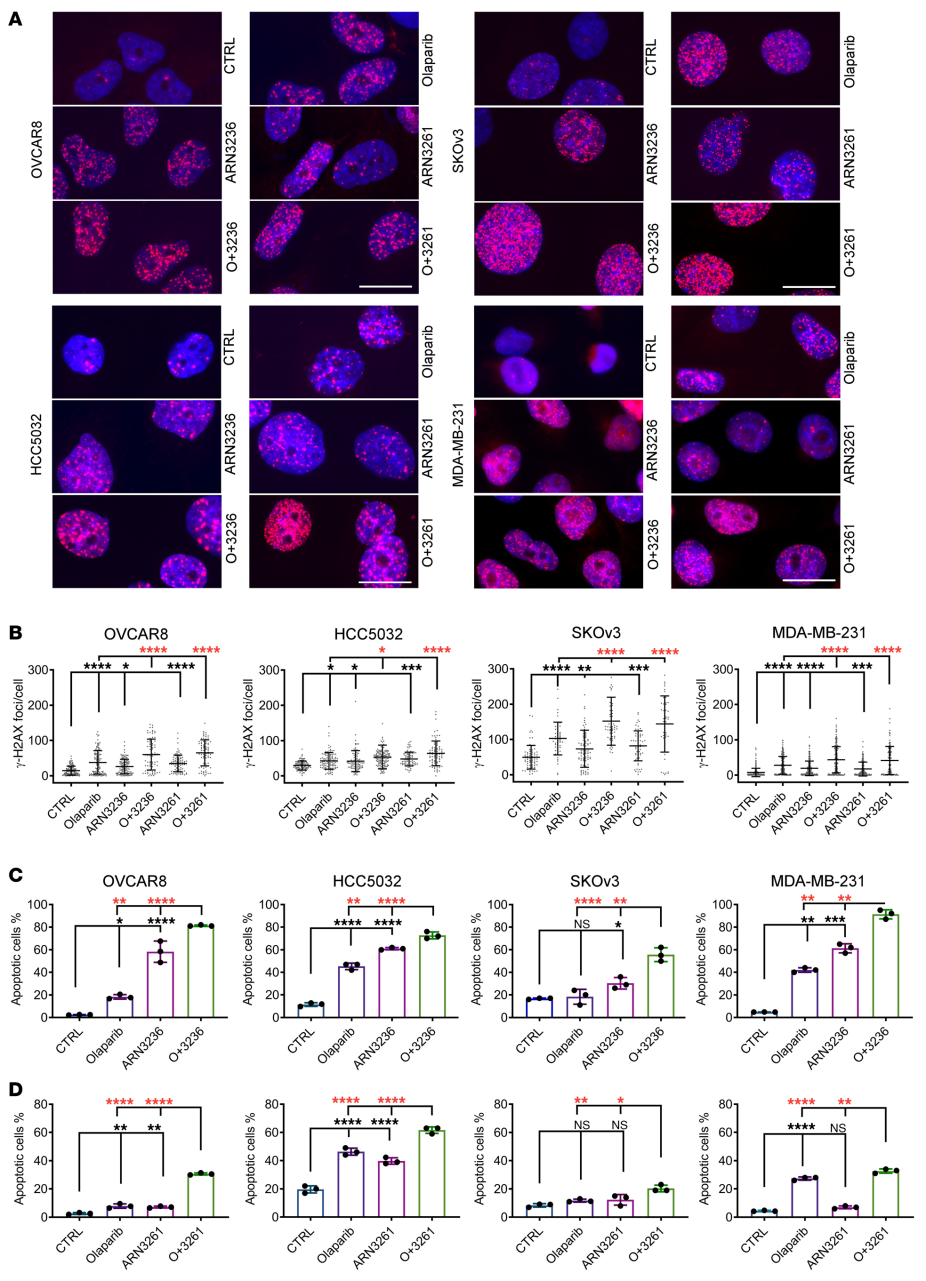
ARN3236 and ARN3261 enhance olaparib-induced apoptosis. (**A** and **B**) Quantification of DNA damage (γ-H2AX). The concentrations of ARN3236, ARN3261, and olaparib were 1 μM, 4 μM, and 2 μM, respectively. Red indicates γ-H2AX and blue (DAPI) indicates nuclear stain. Representative images are presented. Scale bar: 20 μm (**A**). γ-H2AX dots were quantified with Olympus CellSens Dimension software. The middle solid lines indicate the mean. Top and bottom solid lines indicate the SD (**B**). One-way ANOVA and Tukey’s multiple-comparison test were calculated (*P < 0.05; **P < 0.01; ***P < 0.001; ****P < 0.0001). Experiments were from 3 independent experiments with a total of 100–200 cells per treatment. (**C** and **D**) Detection of apoptosis using annexin V/propidium iodide (PI) staining. SKOv3 cells were treated with ARN3236 (8 μM), ARN3261 (5 μM), olaparib (25 μM) alone or combined for 6 days. HCC5032 cells were treated with ARN3236 (1 μM), ARN3236 (3 μM), or olaparib (3 μM) alone or combined for 5 days. OVCAR8 and MDA-MB231 were treated with ARN3236 (6 μM), ARN3236 (6 μM), or olaparib (5 μM) individually or combined for 5 days. Representative data are from 1 experiment with 3 replicates. Experiments were repeated twice with similar results. The columns indicate the mean and the bars indicate the SD. One-way ANOVA and Tukey’s multiple-comparison test were calculated (NS, *P* > 0.05; *P < 0.05; **P < 0.01; ***P < 0.001; ****P < 0.0001).

To confirm whether SIK2 inhibitors induce DNA damage in cancer cells by inhibiting DNA repair, we tested the effect of SIK2 inhibitors on olaparib-mediated induction of DNA DSBs. ARN3236, ARN3261, or olaparib modestly increased levels of both phosphorylation of H2AX (γ-H2AX) and the percentage of tailed DNA, whereas combined treatment of SKOv3, OVCAR8, HCC5032, or MDA-MB-231 cells with ARN3236 or ARN3261 and olaparib increased the levels of γ-H2AX (Figure 5, A and B) and the percentage of tailed DNA (Supplemental Figure 3C) significantly. ARN3261 induced higher levels of γ-H2AX in cancer cells than normal cells (Supplemental Figure 3D). Together, these data are consistent with the possibility that SIK2 inhibition blocked DNA DSB repair. Given that unrepaired DSB can trigger apoptosis, we measured annexin V expression to determine whether the combination of a SIK2 inhibitor and olaparib induced greater levels of apoptosis. ARN3236 or ARN3261 combined with olaparib treatment induced significantly higher levels of apoptosis than either single agent (Figure 5, C and D), consistent with the critical prerequisite of DNA DSB repair for cancer cell survival. Together, these results suggest that preventing DNA DSB repair by SIK2 inhibitors enhances the vulnerability of cancer cells to PARP inhibition.

### SIK2 inhibition decreases phosphorylation of class-IIa HDACs and promoter activity of MEF2 transcription factors.

**Figure 6:**
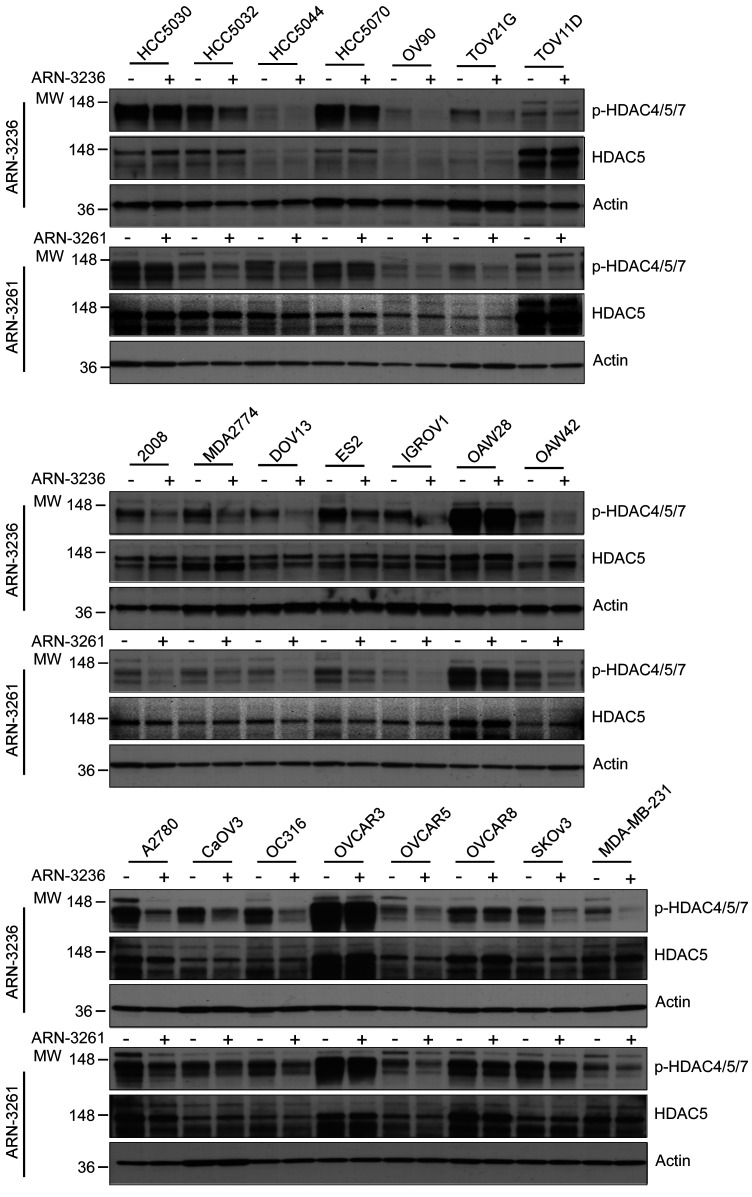
ARN3236 and ARN3261 decrease phosphorylation of HDAC4/5/7. Phosphorylation level of HDAC4/5/7. Twenty ovarian cancer and 2 TNBC cell lines were treated with ARN3236 (4 μM) or ARN3261 (4 μM) for 24 hours. Representative image is from 1 independent experiment. Experiments were repeated twice with similar results.

**Figure 7:**
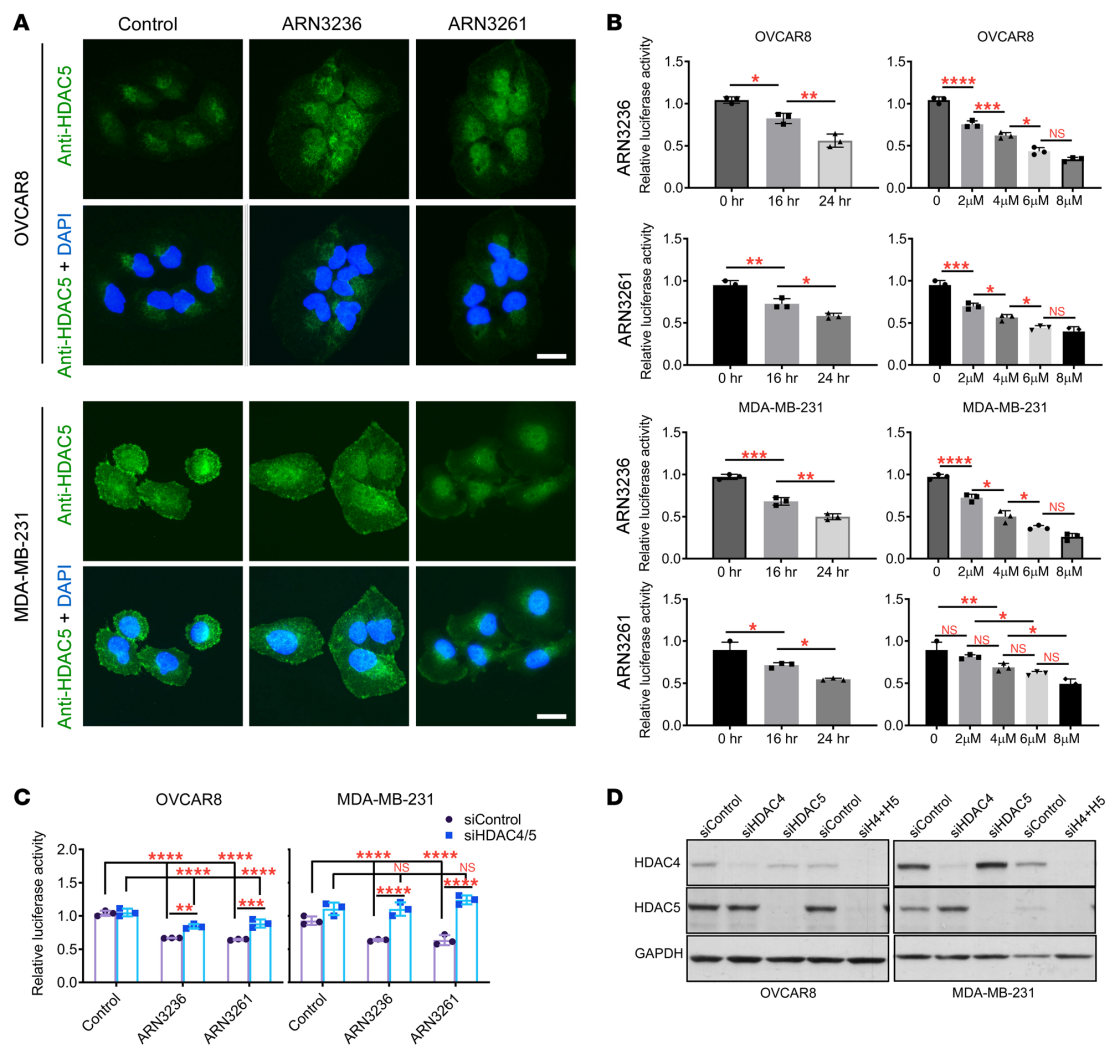
ARN3236 and ARN3261 decrease promoter activity of MEF2 transcription factors. (**A**) Detection of HDAC5 localization with or without SIK2 inhibitors. After overnight incubation, cells were treated with ARN3236 (3 μM) or ARN3261 (5 μM) for 24 hours. Cells were stained with anti-HDAC5 and imaged with fluorescence microscopy for HDAC5 (green) and DAPI (blue). The fluorescence intensity was quantified using ImageJ (Supplemental Figure 4). Scale bar: 20 μm. (**B**) Quantification of MEF2 promoter activity. Cells were transfected with a mixture of a MEF2-responsive luciferase construct and *Renilla* luciferase construct for 24 hours and then treated with ARN3236 (4 μM) and ARN3261 (4 μM) for different intervals or with different doses of inhibitor for 24 hours as indicated. The columns indicate the mean of MEF2 luciferase activity, and the bars indicate the SD. One-way ANOVA and Tukey’s multiple-comparison test were performed (NS, *P* > 0.05; *P < 0.05; **P < 0.01; ***P < 0.001; ****P < 0.0001). Representative data were from 1 independent experiment with 3 technical repeats. Experiments were repeated 2 times. (**C** and **D**) Quantification of MEF2 promoter activity with and without knockdown of HDAC4 and HDAC5 (**C**). Cells were transfected with targeting or control siRNA for 24 hours prior to transfection of a mixture of a MEF2-responsive luciferase construct and *Renilla* luciferase construct. Cells were then treated with ARN3236 (4 μM) or ARN3261 (4 μM) for 24 hours. HDAC4 and HDAC5 siRNA knockdown efficiency was measured by Western blot analysis (**D**). Representative data are from 1 independent experiment with 3 replicates. Experiments were repeated twice with similar results. Two-way ANOVA and Dunnett’s multiple-comparison test were performed (NS, *P* > 0.05; *P < 0.05; **P < 0.01; ***P < 0.001; ****P < 0.0001).

To identify the mechanism(s) by which SIK2 inhibition decreases DNA DSB repair, we tested whether SIK2 inhibitors decrease the phosphorylation of class-IIa HDAC, which controls its nuclear-cytoplasmic shuttling and consequently its association with DNA or DNA-binding transcriptional factors. ARN3236 or ARN3261 significantly decreased the phosphorylation of HDAC4 (Ser246)/HDAC5 (Ser256)/HDAC7 (Ser155) in all the cell lines we tested by Western blotting analysis using an antibody recognizing all 3 phosphorylation sites simultaneously (Figure 6). Next, we investigated whether SIK2 inhibitors increase nuclear localization of HDAC5. SIK2 inhibition increased nuclear localization of HDAC5 judged by increasing nuclear florescence intensity (Figure 7A and Supplemental Figure 4A) and the nuclear fraction of HDAC5 expression (Supplemental Figure 4B). This result raised the possibility that SIK2 inhibition downregulates expression of DNA repair genes by enhancing binding of class-IIa HDAC with DNA-binding transcriptional factors, for which class-IIa HDAC may serve as a transcriptional corepressor complex blocking the expression of MEF2 downstream targets (28, 29). Therefore, we hypothesized that SIK2 inhibition may block MEF transcription factor activity. To test this hypothesis, MEF2 promoter activity was measured using a luciferase reporter assay in ovarian and breast cancer cell lines in the presence and absence of the SIK2 inhibitors ARN3236, ARN3261, or olaparib. SIK2 inhibitors significantly reduced MEF2 promoter activity in a time- and dose-dependent manner (Figure 7B) but olaparib did not (Supplemental Figure 4C). Next, we examined whether SIK2 regulation of MEF2 activity was HDAC4/5 dependent, increasing its binding to MEF2D protein. Knockdown of class-IIa HDAC4/5 with siRNA prevented an ARN3236- or ARN326-mediated decrease of MEF2 promoter activity (Figure 7, C and D), but a decrease in MEF2 promoter activity was not prevented by inhibition of HDAC enzyme activity using TMP195, a selective class-IIa HDAC inhibitor (30) (Supplemental Figure 4D). These observations support the hypothesis that SIK2 inhibition increased nuclear localization of HDAC4/5, blocking MEF2 transcription (Supplemental Figure 4E).

### SIK2 inhibition alters MEF2D transcription factor–mediated downstream signaling.

**Figure 8:**
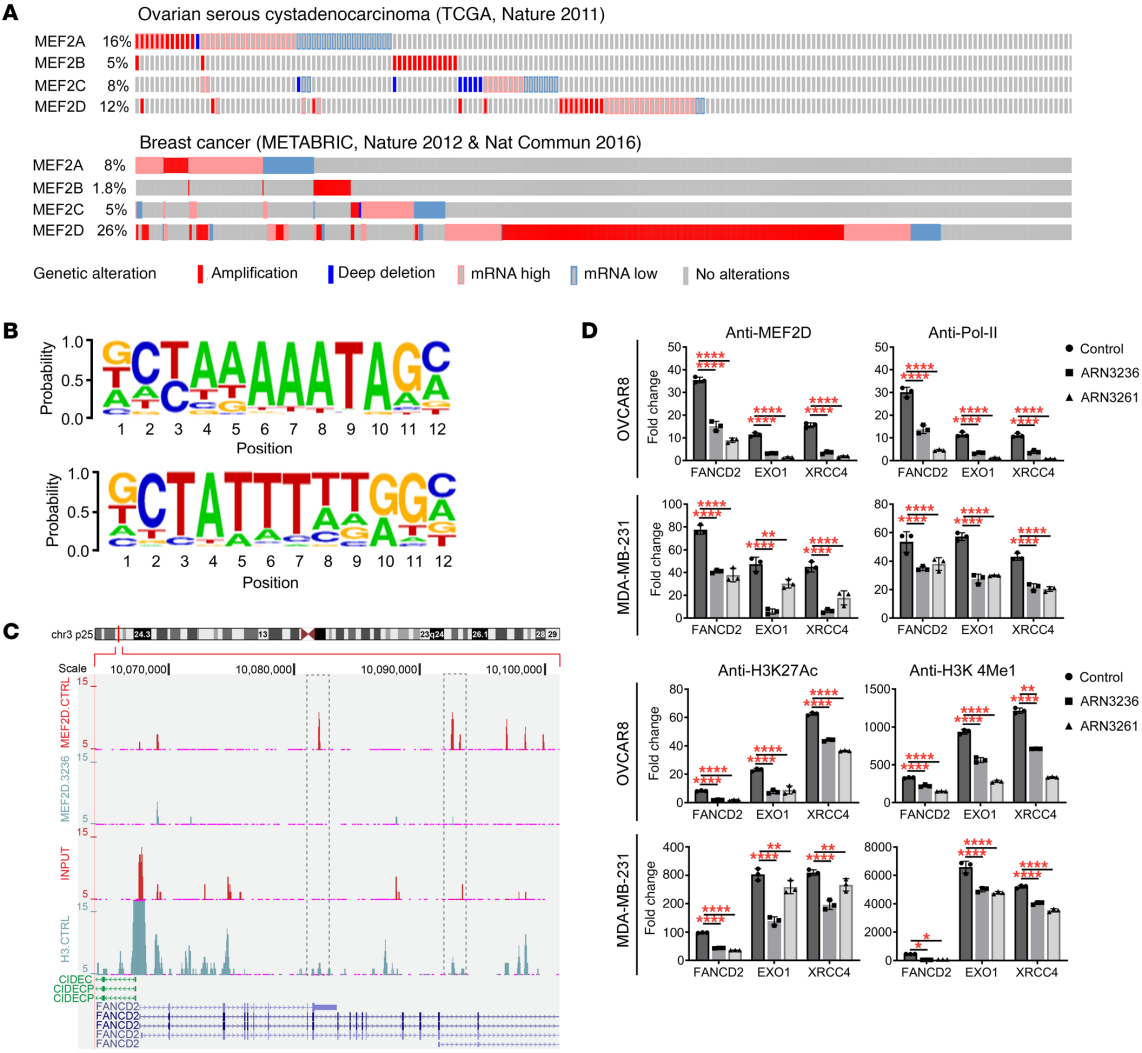
SIK2 inhibition alters MEF2D transcription factor–mediated downstream signaling. (**A**) Alterations affecting MEF2 family genes in ovarian and breast cancer by TCGA analysis. Alterations of MEF2D are found in 12% of ovarian cancer samples (TCGA, 316 samples, ref. 22) and 26% of breast cancer samples (Metabric, 2509 samples, refs. 53, 54) respectively, and the large majority of alterations were amplifications and mRNA upregulations. Data and plots were obtained using cBioPortal (22, 54, 55). (**B**) MEF2D consensus DNA motifs. The MEF2 motif is enriched in MEF2D-binding sites in SKOv3 cells. (**C**) ChIP sequence of anti-MEF2D at the FANCD2 locus in SKOv3 cells treated with and without ARN3236. The dotted line indicates the comparison of chromatin accessibility of the FANCD2 gene between control and ARN3236 treatment. (**D**) ChIP and RT-qPCR analysis of FANCD2, EXO1, and XRCC4 genes. OVCAR8 and MDA-MB-231 cells were treated with and without ARN3236 (6 μM) or ARN3261 (4 μM) for 48–50 hours and then harvested for ChIP analysis with normal IgG, MEF2D, Pol-II, H3K27Ac, or H3KMe1 antibody. ChIP pulldown samples were analyzed by RT-qPCR. The columns indicate the mean of relative fold-changes (fold-change = 2-ΔΔCt, ChIP signal relative to the IgG background signal) and the bars indicate the SD. Two-way ANOVA and Dunnett’s multiple-comparison test were performed (*P < 0.05; **P < 0.01; *****P < 0.0001). Representative data are from 1 experiment with 3 replicates. Experiments were repeated twice with similar results.

To explore the clinical relevance of the MEF2 transcription factors in ovarian cancer and TNBC, we examined alterations in the frequencies of individual MEF2 family members in these tumor types. According to the cBioPortal The Cancer Genome Atlas (TCGA) database, 15%–21% of ovarian and breast cancers contained amplification and mRNA upregulation of *MEF2D* (Figure 8A). We then examined genome-wide binding of MEF2D in SKOv3 ovarian cancer cells using ChIP-Seq. In the genome-wide setting, we identified 73 binding sites of MEF2D and measured a 50% reduction (36 binding sites) in cells treated with ARN3236 (Supplemental Figure 5A). To identify a MEF2D consensus recognition sequence in ovarian cancer cells, de novo motif discovery analysis was performed. A known MEF2 consensus recognition sequence could be detected in 59% (*P* = 1 × 10^–9^) of all random peaks analyzed (Figure 8B). Moreover, motifs containing the consensus sequence for other transcription factors, including Sox15, Usf2, and Sp1, were found at frequencies ranging from 19% to 34%, suggesting that MEF2D can affect expression of downstream targets by associating with the MEF2D DNA-binding site or by interacting with other transcription factors. This result is consistent with previous studies that have suggested MEF2D may function as a transcription factor or enhancer (12, 31, 32). In addition, GO enrichment analysis indicated that MEF2D-bound genes in vehicle-treated SKOv3 cells exhibited significant enrichment in positive regulation of cell differentiation, negative regulation, of cell apoptotic processes and V(D) recombination and positive regulation of DNA repair. By contrast, several MEF2D-bound genes involved in regulation of the TNF-mediated signaling pathway, DNA damage–induced protein phosphorylation, and positive regulation of cell apoptotic process were detected in cells treated with ARN3236 (Supplemental Figure 5B). Moreover, ChIP-Seq analysis indicated that MEF2D binds directly to *FANCD2* (Figure 8C). FANCD2 plays a major role in homology-dependent repair-mediated replication restart and in suppressing new origin firing (33). ChIP-qPCR of *FANCD2* confirmed MEF2D association with the *FANCD2* promoter/enhancer region (Figure 8D and Supplemental Figure 5C). This association was decreased with SIK2 inhibition by ARN3236 or ARN3261 in all 4 cell lines tested (Figure 8D and Supplemental Figure 5C). *EXO1* and *XRCC4* were both downregulated by SIK2 inhibition judged by RNA-Seq (Figure 4A). EXO1 participates in extensive DSB end resection, an initial step in the homologous recombination pathway (34), and XRCC4 is a component of the complex that mediates NHEJ (35). Although *EXO1* and *XRCC4* genes were not associated with MEF2D peaks by ChIP-Seq analysis — possibly due to the poor quality of the anti-MEF2D antibody — potential MEF2D binding sites were identified at the promoter regions of these 2 genes. ChIP-qPCR analysis revealed MEF2D binding to *EXO1* and *XRCC4* promoter/enhancer regions, and MEF2D binding affinities to those targets were significantly decreased with SIK2 inhibition by ARN3236 or ARN3261 in all cell lines tested (Figure 8D and Supplemental Figure 5C). Moreover, MEF2D binding activity to *FANCD2*, *EXO1*, and *XRCC4* was significantly higher in cancer cells than in normal cells, and ARN3261 significantly reduced MEF2D binding activity to *FANCD2*, *EXO1*, and *XRCC4* in cancer cells compared with normal cells (Supplemental Figure 5D). Notably, SIK2 inhibition also reduced H3K27Ac, H3K4me1, and RNA Pol-II binding at the *FANCD2*, *EXO1*, and *XRCC4* promoter/enhancer regions (Figure 8D and Supplemental Figure 5C). Both H3K27Ac and H3K4me1 are the activation marks of enhancers and have regulatory function to increase the transcription of target genes (36–38). PoI-II also is reported to regulate gene transcription by binding to both promoters and enhancers (39, 40). Thus, these data support that FANCD2, EXO1, and XRCC4 are the direct targets of MEF2D and that SIK2 regulates DNA DSB repair by repression of MEF2D transcriptional activity. To evaluate the clinical relevance of the study, we applied Kaplan-Meier survival analysis and found that patients with breast or ovarian cancer with high expression of *FANCD2* and *XRCC4* had poorer overall survival than those with low expression of *FANCD2* and *XRCC4* (Supplemental Figure 6). *EXO1* expression was also positively correlated with survival in breast cancer but not in ovarian cancer (Supplemental Figure 6). These data are consistent with previous reports that overexpression of SIK2 correlates with poor prognosis in patients with ovarian or breast cancer (15, 41).

### Overexpression of MEF2D is sufficient to block SIK2 inhibition–induced DNA damage and growth inhibition.

**Figure 9:**
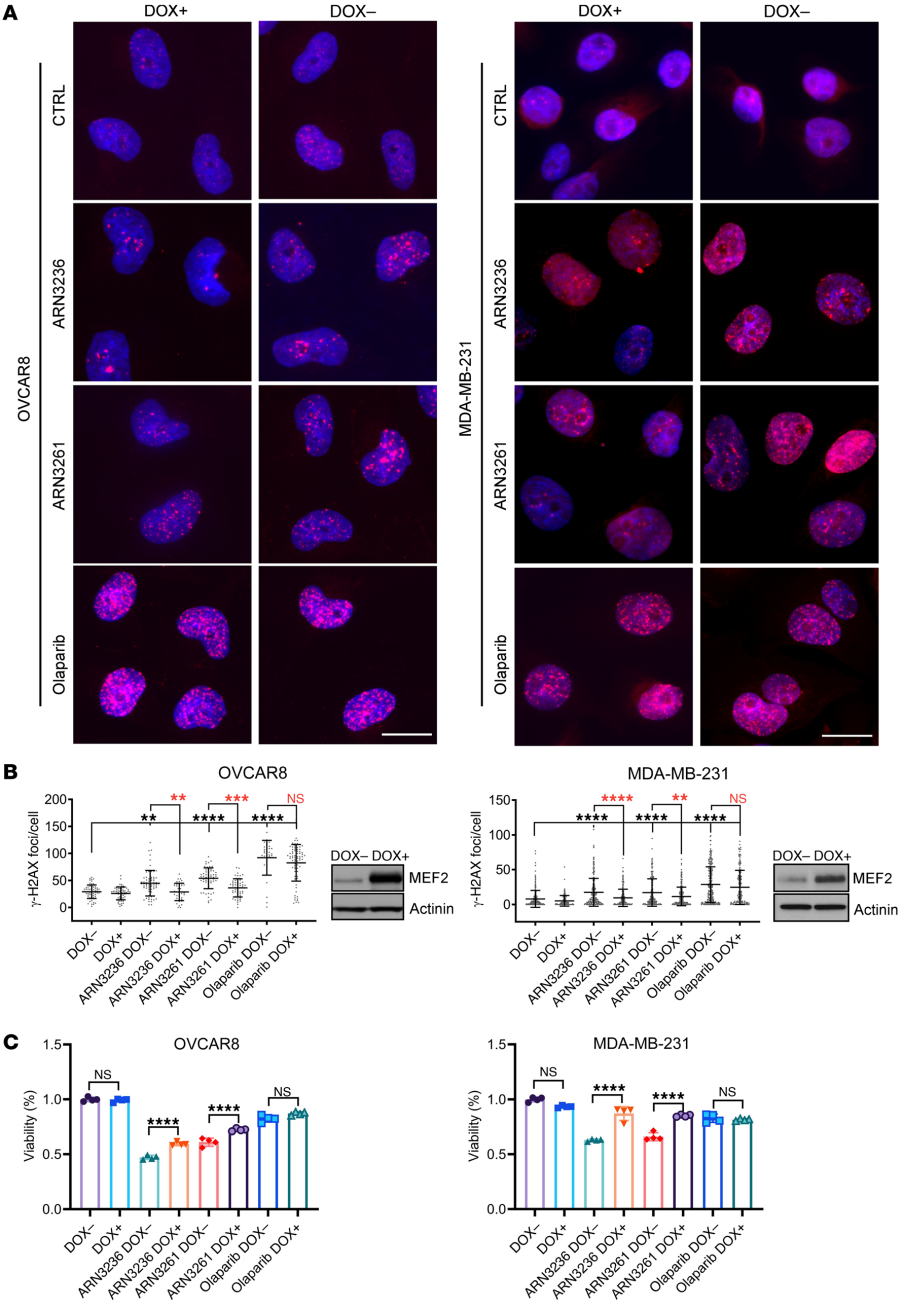
Overexpression of MEF2D is sufficient to block SIK2 inhibition–induced downregulation of FANCD2, EXO1, and XRCC4; DNA damage; and growth inhibition. (**A** and **B**) Forced expression of MEFD2. OVCAR8 and MDA-MB-231 cells with DOX-inducible MEF2D expression were treated with ARN3236 (1 μM), ARN3261 (4 μM), and olaparib (2 μM) in the presence and absence of DOX (1 μg/mL) for 8 hours. DOX was added to culture medium 48 hours prior to inhibitor treatments. Red indicates γ-H2AX and blue (DAPI) indicates nuclear stains. Representative images are presented **A**. Scale bar: 20 μm. γ-H2AX dots were quantified with Olympus CellSens Dimension software. The middle solid lines indicate the mean of fluorescent dots. Top and bottom solid lines indicate the SD. The statistical significance between DOX^–^ and DOX^+^ was calculated with 1-way ANOVA and Tukey’s multiple-comparison test (NS, *P* > 0.05; **P < 0.01; ***P < 0.001; ****P < 0.0001) (**B**). Data were from 3 replicates with a total of 100–200 cells per treatment. Experiments were repeated twice. Determination of MEF2D expression by Western blot analysis (**B**). (**C**) Determination of cell viability in MEF2D DOX-inducible OVCAR8 and MDA-MB-231 cells. DOX-inducible MEF2D sublines of OVCAR8 and MDA-MB-231 were treated with DOX and without DOX for 24 hours, and then treated with ARN3236 (2 μM), ARN3261 (4 μM), and olaparib (4 μM) for 72 hours. The statistical significance between DOX^–^ and DOX^+^ was calculated with 1-way ANOVA and Tukey’s multiple-comparison test. NS, *P* > 0.05; ****P < 0.0001. Representative data were from 1 experiment with 4 replicates. Experiments were repeated twice with similar results.

As described above, SIK2 inhibition blocks HDAC4/MEF2-mediated DNA DSB repair by downregulating the expression of critical factors participating in this process. To test whether MEF2D downregulation was sufficient to explain the effects of SIK2 inhibition on DNA DSB repair and whether overexpression of MEF2D will rescue SIK2 inhibitor–mediated DNA damage and growth inhibition, OVCAR8 and MDA-MB-231 doxycycline-inducible (DOX-inducible) stable cell lines expressing MEF2D were generated. When MEF2D expression was induced by DOX treatment, γ-H2AX foci were significantly decreased in the cells treated with either ARN3236 (*P* < 0.001 in MDA-MB-231 and *P* < 0.0001 in OVCAR8 cells) or ARN3261 (*P* < 0.0001 in both MDA-MB-231 and OVCAR8 cells) but not olaparib compared with uninduced cells with no DOX treatment in both the OVCAR8 (*P* = 0.4514) and MDA-MB-231 (*P* = 0.3511) cell lines (Figure 9, A and B). These data confirmed a role for MEF2D in promoting cancer survival by decreasing DNA damage in cancer cells. In addition, when viability was measured, induction of MEF2D partially rescued toxicity from ARN3236 or ARN3261, but not from olaparib to cells with MEF2D induction (Figure 9C). Together, these results suggest that SIK2 inhibitors enhance the vulnerability of cancer cells to olaparib, not only by inhibiting PARP enzyme activity but also by blocking the class-IIa HDAC/MEF2D–mediated DNA repair function.

### Coadministration of SIK2 inhibitor and olaparib is synergistic in vivo.

**Figure 10:**
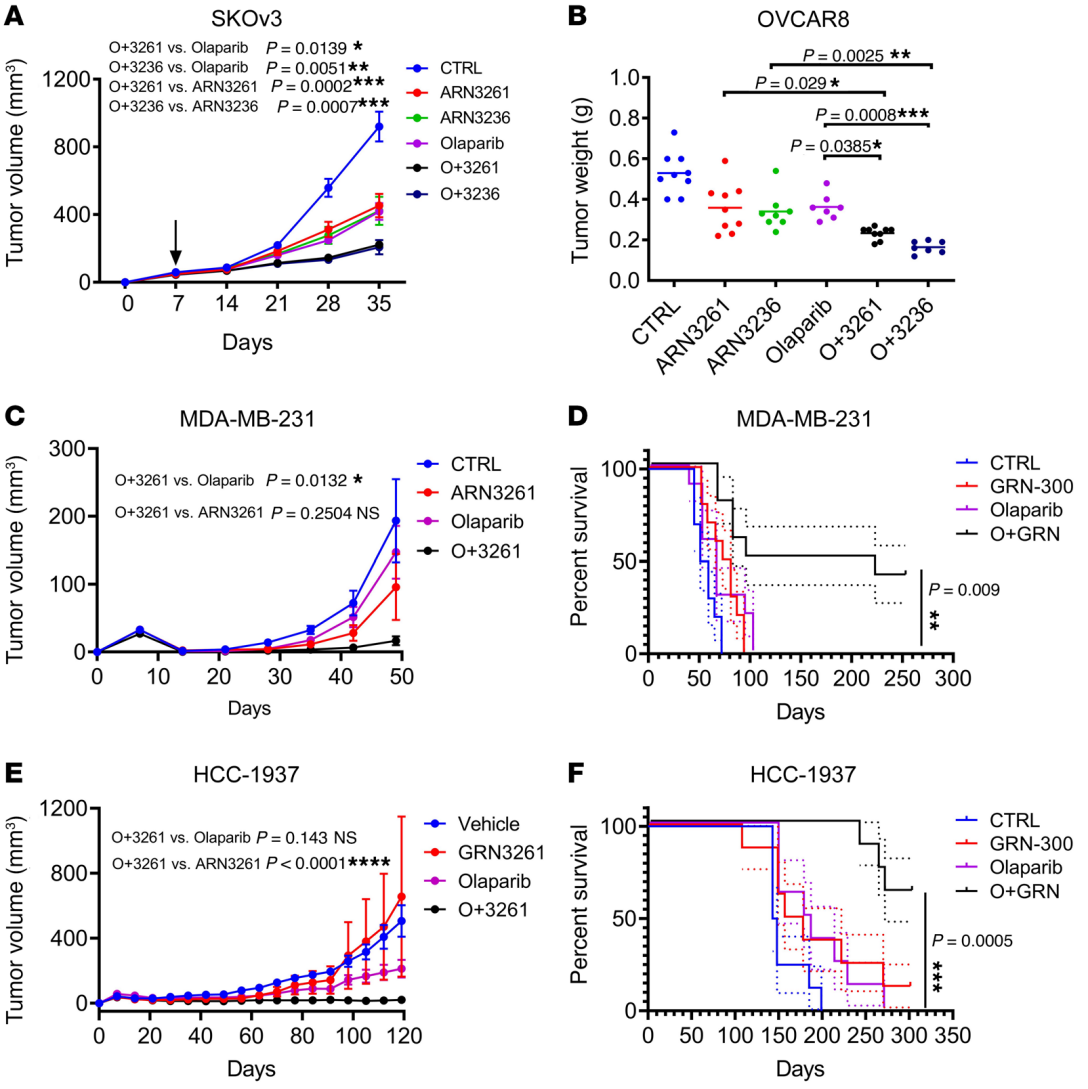
Coadministration of SIK2 inhibitor and olaparib synergistically inhibits xenograft growth. (**A** and **B**) Tumor growth and tumor weight of ovarian cancer xenografts in female athymic *nu/nu* mice after treatment with a single agent or combination (*n* = 10). Tumor growth by tumor volume (**A**) or tumor weight (**B**) under different treatments plotted as mean ± SD. One-way ANOVA and Tukey’s multiple-comparison test were performed (*P < 0.05; **P < 0.01). Both experiments were performed once. (**C** and **D**) Tumor growth of MDA-MB-231 cells and survival of tumor-bearing mice. Tumor-bearing mice were randomized into 4 treatment groups (*n* = 10) after 7 days of tumor growth. Mice were treated with a single agent or combination for 6 weeks. Experiments were repeated 2 times. Tumor growth was evaluated from the start of treatment until tumors reached 1500 mm^3^. One-way ANOVA and Tukey’s multiple-comparison test were performed for tumor growth. Survival was evaluated with ethical endpoints. Survival curves were generated by GraphPad Prism 6. A log-rank test was performed for comparison of survival (NS, *P* > 0.05; *P < 0.05; **P < 0.01; ***P < 0.001). (**E** and **F**) Tumor growth of HCC-1937 TNBC cells and survival of tumor-bearing mice. Tumor-bearing mice were randomized into 4 treatment groups (*n* = 8) after 7 days of tumor growth. Mice were treated with a single agent or combination for 4 weeks. The experiment was performed once. Tumor growth was evaluated from the start of treatment until tumors reached 1500 mm^3^. One-way ANOVA and Tukey’s multiple-comparison test were performed for tumor growth. Survival curves were generated as above. A log-rank test was performed for comparison of survival (NS, *P* > 0.05; *P < 0.05; **P < 0.01; ***P < 0.001).

Based on enhancement of PARP inhibitor activity by SIK2 inhibition in cell culture, we tested whether the addition of SIK2 inhibitors could promote the PARP inhibitor response in vivo. When the *BRCA*-proficient SKOv3 cell line was s.c. injected into mice, treatment with ARN3236, ARN3261, or olaparib alone significantly inhibited tumor growth compared with a vehicle control (Figure 10A). The combination of ARN3236 plus olaparib or ARN3261 plus olaparib produced greater inhibition of tumor growth than did either single agent (Figure 10A). Another *BRCA*-proficient OVCAR8 ovarian cancer cell line was i.p. injected into mice that were treated as described for the SKOv3 xenograft model. ARN3236 or ARN3261 in combination with olaparib significantly inhibited OVCAR8 tumor growth to a much greater degree than either single agent (Figure 10B). In the OVCAR8 i.p. xenograft model, ARN3236 or ARN3261 in combination with olaparib decreased formation of ascites (Supplemental Figure 7B). Moreover, the combination was well tolerated, with no significant weight loss compared with vehicle control in both SKOv3 (Supplemental Figure 7A) and OVCAR8 (Supplemental Figure 7B) models. In addition, the OC316 (heterozygous *BRCA2* mutated) ovarian cancer xenograft model was used to extend results observed with SKOv3 and OVCAR8 xenografts. Similar results were observed in the OC316 xenograft model (Supplemental Figure 7, C–E). More importantly, the ARN3261 and olaparib combination prolonged survival compared with either agent alone, with tumor regression in 2 out of 10 xenografts (*P* = 0.027) (Supplemental Figure 7D). To demonstrate relevance to breast cancer, we studied xenografts with both a *BRCA*-proficient TNBC cell line model MDA-MB-231 and a *BRCA*-deficient TNBC cell line model HCC1937. To reflect the original microenvironment, MDA-MB-231 and HCC1937 cells were implanted directly into the mammary fat pad of female nude mice. One week after cell inoculation, mice were treated with the single agents ARN3261 or olaparib or the combination, and tumor volume was measured at the indicated intervals (Figure 10, C and E). After treatment with either single agent, ARN3261 or olaparib, tumor burden decreased compared with the vehicle control; however, the combined treatment inhibited tumor volume around day 28 (Figure 10, C and E) and induced tumor regression and prolonged survival in 4 of 10 mice (*P* = 0.009) (Figure 10D) and 5 of 8 mice (*P* = 0.0005) (Figure 10F) in MDA-MB-231 and HCC1937 cancer cell models, respectively.

**Figure 11:**
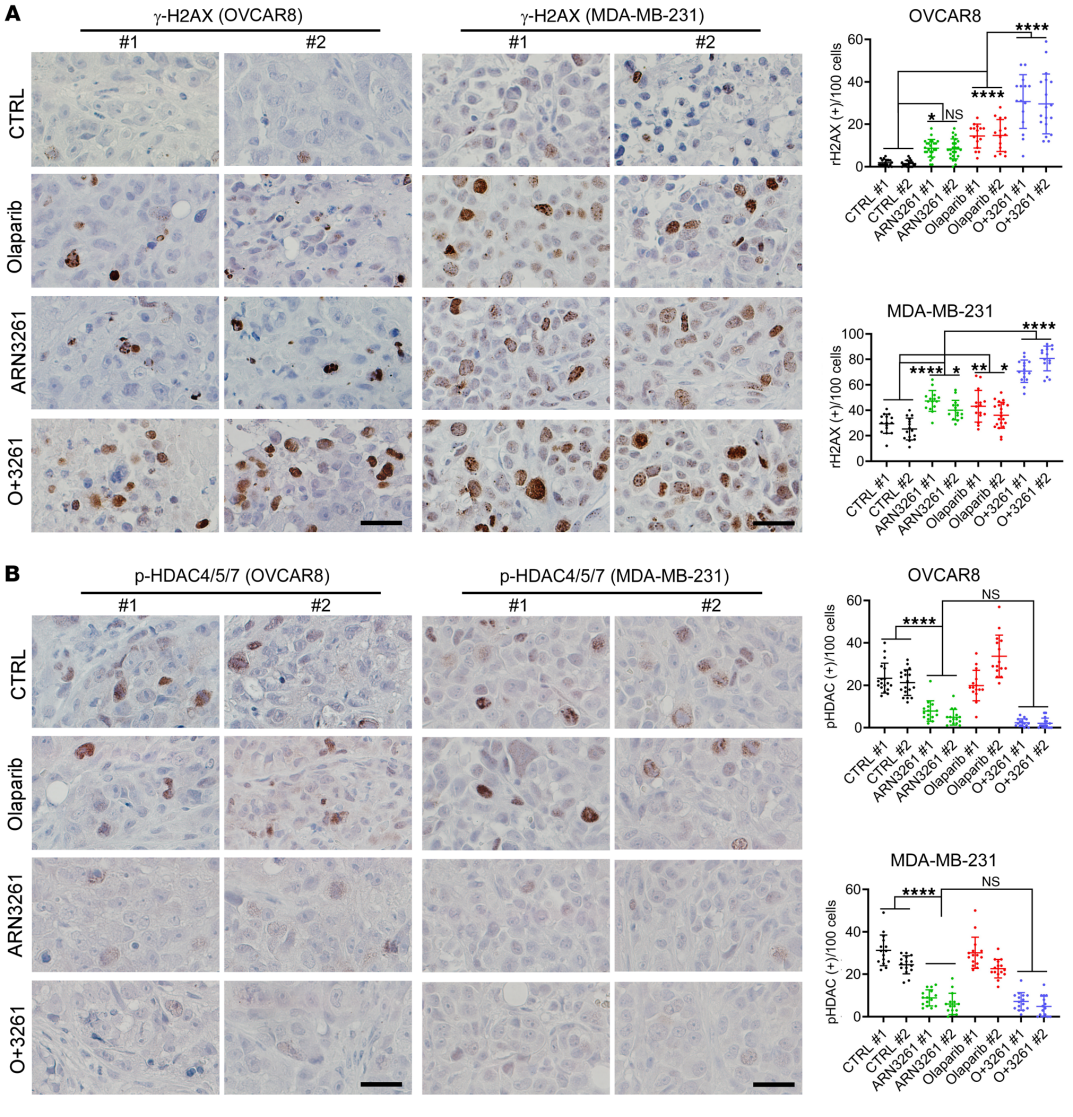
Coadministration of SIK2 inhibitor and olaparib increases γ-H2AX and decreases phosphorylation of HDAC4/5/7 in OVCAR8 and MDA-MB-231 tumor xenografts. Representative images of IHC with indicated antibodies, γ-H2AX (**A**) and p-HDAC4/5/7 (**B**), from mouse tumor tissues. Scale bar: 50 μm. Positive cells per 100 cancer cells were counted and 1-way ANOVA with Tukey’s multiple-comparison test were performed (NS, *P* > 0.05; *P < 0.05; **P < 0.01; ***P < 0.001; ****P < 0.0001). #1 indicates mouse #1 and #2 indicates mouse #2 from 1 experiment.

Tumors growing as xenografts were collected for histology with H&E and IHC staining. Routine H&E staining detected high-grade ovarian cancer in ovarian cancer xenograft models and breast cancer morphology in the breast cancer xenograft model, respectively. IHC of OVCAR8 and MDA-MB-231 xenograft tumors at study termination recapitulated in vitro studies. ARN3261 increased nuclear γ-H2AX staining, which was further increased by treatment with ARN3261 in combination with olaparib (*P* < 0.0001) (Figure 11A). Nuclear p-HDAC4/5/7 staining was decreased in ARN3261-treated tumors (*P* < 0.0001) but not in olaparib-treated tumors (Figure 11B). These data are consistent with the notion that SIK2 inhibition enhances olaparib sensitivity through increasing nuclear localization of class-IIa HDACs, decreasing MEF2D-mediated expression of DNA repair genes, and increasing DNA damage. Taken together, these preclinical models demonstrated that SIK2 provides a target that could contribute to the care of patients with HGSOC or TNBC.

## Discussion

Our study found that SIK2 inhibition impeded DNA DSB repair, increasing DNA damage and synergistically enhancing sensitivity of HGSOC and TNBC to PARP inhibitors in cell culture and xenograft models. Increased expression of γ-H2AX, a DNA damage marker, was observed in ovarian and breast cancer cells but not in normal ovarian and breast epithelial cells. Synergistic activity was noted in *BRCA* mutant and wild-type cancers. Decrease of PARP enzyme activity and phosphorylation of class-IIa HDAC4/5/7 were necessary and sufficient for the synergy observed between SIK2 inhibitors and PARP inhibitors for downregulating cancer cell growth in ovarian and breast cancer cell lines and xenografts. Inhibition of the phosphorylation of class-IIa HDAC4/5/7 by ARN3236 or ARN3261 SIK2 inhibitors a) abolished class-IIa HDAC4/5/7–associated transcriptional activity of MEF2, b) decreased MEF2D binding to regulatory regions with high-chromatin accessibility in DNA repair genes, and c) repressed expression of critical genes in the DNA DSB repair pathway. Decreased expression of *FANCD2*, *EXO1*, and *XRCC4* due to SIK2 inhibition likely contributes to PARP inhibitor sensitivity through a MEF2D-dependent mechanism.

SIK2 inhibition decreased phosphorylation of class-IIa HDACs and increased nuclear localization of class-IIa HDAC proteins. Phosphorylation of class-IIa HDACs controls their signaling-dependent nucleocytoplasmic shuttling. Under basal conditions, class-IIa HDACs are unphosphorylated and located in the nucleus, where they are recruited to their target genes through interaction with transcription factors, enabling their transcriptional repressive function. Class-IIa HDACs become phosphorylated in response to specific signals, leading to disruption of the interaction with transcription factors, their export to the cytoplasmic compartment, and derepression of their targets (42, 43). A member of class-IIa HDACs was thought to be a component of the DNA damage response, recruited to the same dots or repair foci together with 53BP1, which is vital in promoting NHEJ (44). We demonstrated that SIK2 regulation of the MEF2D-mediated DNA repair pathway depends upon SIK2-mediated phosphorylation of class-IIa HDACs. Thus, class-IIa HDACs appear to be the key regulators of the synergy observed between SIK2 inhibitors and PARP inhibitors.

MEF2 transcription factors serve diverse functions in a wide range of tissues and have been implicated in several diseases (31). The spectrum of genes regulated by MEF2 in different cell types depends upon extracellular signaling and on cofactor interactions that modulate MEF2 activity. The MEF2 domain is also involved in interactions with coactivators and corepressors. Corepressors that are thought to associate with the MEF2 domains of all MEF2 family proteins include class-IIa HDAC4, HDAC5, HDAC7, and HDAC9 (45, 46). According to the cBioPortal database, 6% to 21% of ovarian serous cystadenocarcinomas, invasive breast cancer, lung squamous cell and adenocarcinomas, uterine endometrioid carcinomas, stomach adenocarcinomas, adrenocortical carcinomas, esophageal carcinomas, bladder urothelial carcinomas, and pancreatic adenocarcinomas contain amplified *MEF2* genes. Our study documents that *MEF2* genes may act as oncogenes by regulating expression of genes involved in DNA DSB repair in ovarian and breast cancers. SIK2 inhibition decreased *MEF2* gene promoter activity and repressed expression of critical genes in the DNA DSB repair pathway, supporting the notion that ARN3236 and ARN3261 enhance sensitivity to PARP inhibitors by decreasing MEF2’s oncogenic function.

Synergetic interaction of SIK2 inhibitors and PARP inhibitors was observed with 3 structurally distinct PARP inhibitors (rucaparib, niraparib, and talazoparib) that have differential PARP trapping potential (25). Combinations of SIK2 inhibitors with PARP inhibitors of higher PARP trapping potential (talazoparib) and with lower PARP trapping activity (olaparib) produced similar combination indices, consistent with comparable synergy. Measurement of PARP enzyme activity indicated that the SIK2 inhibitors enhanced the effect of olaparib by further decreasing PARP enzyme activity in cancer cells with detectable PARP protein levels. Furthermore, 2 different SIK2 inhibitors demonstrated synergy with PARP inhibitors, consistent with on-target effects of the SIK2 inhibitor. PARP inhibitors elicit significant responses in *BRCA1* or *BRCA2* mutation carriers with breast, ovarian, prostate, and pancreatic tumors (47, 48). SIK2 inhibitors enhance olaparib sensitivity and inhibit tumor cell growth in both *BRCA1/2* mutant and wild-type cancer cells. Thus, developing new strategies to enhance PARP inhibitor sensitivity and expand the utility of PARP inhibitors to DNA DSB repair–competent tumors is crucial.

This study has a number of limitations. We have documented that decreased expression of *FANCD2*, *EXO1*, and *XRCC4* due to SIK2 inhibition likely contributes to PARP inhibitor sensitivity through a MEF2D-dependent mechanism. MEF2, however, regulates the expression of many molecules, and there may be additional effects of MEF2D that contribute to sensitization to PARP inhibitors in cooperation with downregulation of *FANCD2*, *EXO1*, and *XRCC4*. Previously, we showed SIK2 inhibition by ARN3236 significantly inhibits AKT signaling (18) and PI3K. In the current study, we found that neither siRNA-mediated knockdown of PI3K nor knockdown of AKT failed to block ARN3261-induced downregulation of p-HDAC4/5/7 (unpublished observations). Because knockdown of AKT expression by RNAi was incomplete, it was not possible to rule out that SIK2 inhibition of AKT signaling might contribute to the enhancement of PARP inhibitor sensitivity in ovarian and breast cancers through a class-IIa HDACs/MEF2D–independent pathway. In addition, we have reported that ARN3261 enhanced cytotoxic chemotherapy with carboplatin in ovarian cancer cells and xenografts (19) and showed that ARN3261 significantly decreased the IC_50_ of several other standard chemotherapy drugs, including olaparib, paclitaxel, carboplatin, cisplatin, etoposide, doxorubicin, and topotecan in OVCAR8 and MDA-MB-231 cells. Greater decreases of IC_50_ were, however, seen when ARN3261 was combined with olaparib, carboplatin, and cisplatin (11.2- to 28.6-fold change in OVCAR8 cells and 3.0- to 4.9-fold change in MDA-MB-231 cells) compared with ARN3261 combined with paclitaxel, etoposide, doxorubicin, and topotecan (3.0- to 4.9-fold change in OVCAR8 cells and 2.0- to 2.4-fold change in MDA-MB-231 cells) (unpublished observations). However, ARN3261 enhanced the antitumor effect of other chemotherapy drugs in vivo, which warrants further study. PARP inhibitors represent a promising treatment strategy, although the majority of patients with ovarian cancer who receive PARP inhibitors will inevitably experience resistance to them (49). Combination therapy with ARN3261 and olaparib synergistically decreased cell viability at the doses optimized for synergy using 2 PARP inhibitor–resistant ovarian cancer cell lines (unpublished observations). RNA-Seq analysis demonstrated a global increase in DNA repair gene expression in olaparib-resistant cells compared with olaparib-sensitive cells, and combined treatment significantly downregulated the expression of DNA repair genes in both olaparib-sensitive and -resistant cells (unpublished observations), suggesting that transcriptional regulation of gene expression might contribute to ARN3261-mediated enhancement of olaparib sensitivity in olaparib-resistant cells. Nonetheless, further studies are needed to confirm the mechanisms of action of ARN3261 on PARP inhibitor resistance. Finally, although our in vivo data strongly support efficacy and low toxicity, we have not yet demonstrated the activity and tolerability of a SIK2 inhibitor and PARP inhibitor combination in patients.

Taken together, SIK2 inhibition decreases PARP enzyme activity and the expression of *FANCD2*, *EXO1*, and *XRCC4*, suggesting that the combination of a SIK2 inhibitor and PARP inhibitor has the potential to increase the magnitude and duration of PARP inhibitor activity in patients with different cancers. Thus, future clinical trials could be designed to determine whether the combination will benefit patients with ovarian cancer, TNBC, or prostate cancer. Our animal studies, particularly those with olaparib and ARN3236/ARN3261, did not show significant toxicity based on weight loss. Preclinical toxicology studies in rodents and dogs showed no hematological toxicity, which is particularly important for combination with PARP inhibitors. The potential for tolerability in patients is further supported by the lack of synergism of the combination in the normal cell lines. ARN3261 (renamed GRN300) is currently being evaluated in a phase I trial to find the maximum tolerated dose of GRN300 alone and in combination with paclitaxel for patients with ovarian cancer. Assessing the combination of a PARP inhibitor and SIK2 inhibitor in the clinical setting should therefore be prioritized to optimize the use of these compounds and to maximize patient benefit.

## Methods

### Study design.

The objective of this study was to define the effect of SIK2 inhibitors (ARN3236 and ARN3261) on cancer cell growth in ovarian cancer and TNBC as well as to explore mechanisms underlying the synergy between SIK2 and PARP inhibition. We demonstrated that SIK2 inhibition synergistically enhanced PARP inhibitor activity in a variety of ovarian cancer and TNBC cell lines and xenograft models. In vitro experiments were performed in biological triplicate unless otherwise stated. Sample sizes were determined on the basis of previous experience and were sufficient to detect statistically significant differences between treatments. For in vivo experiments, mice were randomly assigned to treatment groups. Experiments were not blinded. Study groups were followed until individual tumor volume reached 1500 mm^3^, at which point euthanasia was indicated in accordance with IACUC protocols.

### Cell lines.

Cell lines used in this manuscript are listed in Supplemental Table 1. The identity of all cell lines was confirmed with short tandem repeat DNA fingerprinting in the MD Anderson Cancer Center Characterized Cell Line Core (supported by NIH National Cancer Institute [NCI] P30CA016672). All cell lines were maintained in an incubator with 5% CO_2_ at 37°C. Freedom from mycoplasma contamination was tested periodically with a Universal Mycoplasma Detection kit from ATCC (30-1010K).

### Viability assays.

Cell viability was determined using CellTiter-Glo Luminescent Cell Viability assay (Promega, G9241). First, 2000–4000 cells were plated in 96-well plates and treated with a SIK2 inhibitor (ARN3236 or ARN3261) and a PARP inhibitor (rucaparib, niraparib, olaparib, or talazoparib) alone or combined in serial dilutions 24 hours after seeding. After 5 days of incubation, media were removed and a mixture of 30 μL of CellTiter-Glo reagent and 60 μL of culture media was added to each well. Luminescence was measured on a Synergy2 microplate reader (BioTek) after 10 minutes of shaking. Dose-response experiments were plotted and IC_50_ values were calculated using nonlinear curve-fitting with normalized response and variable slope using GraphPad Prism 8. Drug interaction of the 2-drug combination was evaluated using a constant ratio. Data were processed and a combination index calculated using CalcuSyn 2.0 software (BIOSOFT). Combination index less than 1 indicates synergism, combination index equal to 1 indicates an additive effect, and combination index greater than 1 indicates antagonism.

### Clonogenic assays.

Individual cells were seeded in 6-well plates in triplicate at a density of 200, 400, or 600 cells/well depending on doubling time. Cells were treated with 1 or 2 agents at different concentrations 1 day after seeding. Cells were grown up to 2 weeks until visible colonies were formed. Culture media with different treatments were refreshed every other day. At the conclusion of the experiment, cells were washed twice with PBS, fixed in 0.1% Brilliant blue R with 10% v/v acetic acid and 30% v/v methanol for 1 minute, and washed with tap water until the intercolony background was clear. Images were captured using a FluoChem E Imager. Clones with more than 50 cells were counted.

### PARP trapping assays.

Chromatin extraction was performed as described by Muvarak and colleagues using a subcellular protein fractionation kit (Thermo Fisher Scientific, 78840) (50). Briefly, pellets were first lysed in membrane extraction buffer. Nuclei were then lysed in nuclear extraction buffer to isolate a nuclear soluble fraction. The remaining chromatin (nuclear insoluble) fraction was washed once with nuclear extraction buffer, and then digested with 300 units of micrococcal nuclease to release chromatin-bound proteins. PARP binding in the chromatin fraction (indicative of PARP trapping) was assayed by Western blot analysis of the chromatin cell fraction against the PARP antibody (BD Biosciences, 551025) (51).

### PARP enzyme activity assay.

PARP enzyme activity was measured using a PARP universal colorimetric assay kit (R&D Systems, 4677-096-K). Cells from different ovarian cancer cell lines were plated and treated with ARN3236 (6 μM) or ARN3261 (4 μM), olaparib (0.05 μM), or a combination of both for 26 hours. Cell lysates were collected using cell extraction buffer. The biotinylated poly(ADP-ribose) deposited by PARP-1 in cell lysates onto immobilized histones in a 96-well plate was detected. Streptavidin-HRP (biotin-binding protein) and a colorimetric HRP substrate were added to produce relative absorbance that correlated with PARP-1 activity.

### ChIP and RT-qPCR analysis.

OVCAR8, MDA-MB-231, SKOv3, OVCAR8-SIK2 KO, or SKOv3-SIK2 KO cells (2 million) were cultured on a 150 cm plate and treated the next day either with vehicle control or with ARN3236 (4 μM) or ARN3261 (5 μM) for 48 hours. ChIP assays were performed using the Magna ChIP A kit (MilliporeSigma, 17-610). Detailed procedures and analysis are included in Supplemental Methods. Purified and enriched DNA was quantified using RT-qPCR with the following primers: *FANCD2* forward, 5′ CGTGAAGTCTGGCTTAGGATTAG 3′ and reverse, 5′ CCCTTCTTCAATACTTCCCTACC 3′; *EXO1* forward, 5′ GGTCTGGCCTAAGGTTTCTTC 3′ and reverse, 5′ CAGTTCACGCTGGGTTCTT 3′; and *XRCC4* forward, 5′ GCAGTCTTCCTAGTCTCAACTG 3′ and reverse, 5′ TTGCCCTTCTAGGAGCTTAATG 3′. RT-qPCR was performed using iTaq Universal SYBR Green Supermix (Bio-Rad, 172-5124) in a CFX Connect RT-qPCR (Bio-Rad). Thermal cycling condition was as follows: 94°C for 10 minutes, followed by 40 cycles of 94°C for 20 seconds and 60°C for 60 seconds. Analysis of qPCR data was calculated using the fold enrichment method (the ChIP signals are divided by the IgG antibody signals, 2^–ΔΔCt^).

### mRNA-Seq, ChIP-Seq, and analysis.

Sequencing was performed by the Sequencing and Microarray Facility at MD Anderson Cancer Center. Detailed procedures and analysis are included in Supplemental Methods. mRNA-Seq data were submitted to National Center for Biotechnology Information’s BioProject (PRJNA797060).

### Immunoblot.

Cells were incubated with and without treatment for the intervals indicated, and then cells were incubated in lysis buffer (50 mM HEPES, pH 7.0; 150 mM NaCl; 1.5 mM MgCl_2_; 1 mM EGTA; 10% glycerol; 1% Triton X-100; 50 mM NaF, 1 mM Na_3_VO_4_; 1 mM PMSF; 10 μg/mL leupeptin; and 10 μg/mL aprotinin) on ice for 30 minutes. Lysates were centrifuged at 15,000*g* at 4°C for 15 minutes, and supernatants were collected. To prepare subcellular fractions of nuclear soluble and chromatin-bound material, cells were treated with indicated drugs and then collected by scraping. For fractionation, we used a Subcellular Protein Fractionation kit (Thermo Fisher Scientific, 78835) following the manufacturer’s instructions. The protein concentration was assessed using a bicinchoninic acid protein assay (Thermo Fisher Scientific, 23228). The proteins were separated by SDS-PAGE and transferred to PVDF membranes (Thermo Fisher Scientific, 88518). After being blocked with 5% BSA in Tris-buffered saline with 0.1% Tween 20 detergent, the membranes were incubated with primary antibodies at 4°C overnight, followed by 1:2000 HRP-conjugated secondary antibody (Thermo Fisher Scientific, goat anti-mouse 31439 and goat anti-rabbit 31463) for 40–60 minutes at room temperature. Bands were visualized using an ECL Western blotting substrate (PerkinElmer, NEL 104001EA). SIK2 (catalog 6919), p-HDAC4/5/7 (catalog 3443), HDAC5 (catalog 20458), HDAC4 (catalog 5392), and actin (catalog 4967) antibodies were purchased from Cell Signaling Technology. GAPDH (catalog MAB374) antibody and actin (catalog MAB110) were from MilliporeSigma. PARP (catalog 551052) and MEF2D (catalog 610775) antibodies were from BD Pharmingen. Lamin A/C (sc-6215) antibody was from Santa Cruz Biotechnology. Actinin (CBL-231) antibody was purchased from Chemicon, and the α-tubulin (T9026) antibody was from MilliporeSigma.

### RNA extraction and RT-qPCR analysis.

Cells were treated with and without ARN3236 or ARN3261 for 72 hours and lysed in TRIzol (Thermo Fisher Scientific, 15596026). Total RNA was extracted using RNeasy Kit (Qiagen, 217004) according to the manufacturer’s instructions. cDNA was synthesized from 2 μg of RNA using the Superscript II First Strand Synthesis Kit (Invitrogen, 11904-018). RT-qPCR was performed using CFX Connect Real-time System (Bio-Rad) in a total volume of 20 μL, which included 10 μL of 2× SsoAdvanced Universal PCR master (PCR primers are included) and 5 ng of cDNA. Thermal cycling conditions were as follows: 95°C for 2 minutes followed by 40 cycles of 95°C for 5 seconds and 60°C for 30 seconds. PrimePCR custom plates (96 well) that contained 2× SsoAdvanced Universal PCR Master Mix and PCR primers were custom ordered from Bio-Rad. Data were analyzed by the ΔΔCt method using GAPDH as a housekeeping gene.

### Establishment of OVCAR8 and SKOv3 SIK2 CRISPR/Cas9 KO cell lines.

OVCAR8 and SKOv3 SIK2 KO ovarian cancer cell lines were established using CRISPR/Cas9 technology as described previously (52). Briefly, a plasmid with GFP containing Cas9 and the sgRNA expression were transfected into cancer cells. CRISPR-mediated KO was performed using guide RNAs targeting exon 2 (AATAATCGATAAGTCTCAGC) and exon 4 (GATTTTCAGCTTTGAGGTCA). Transfected cells were isolated by FACS for single-cell culture 2–3 days after transfection, and then the cells were expanded and harvested for detection of the protein expression using Western blot analysis.

### Establishment of OVCAR8 and MDA-MB-231 MEF2D inducible cell lines.

OVCAR8 and MDA-MB-231 cells were infected with pLV(Exp)-Neo-CMV>tTS/rtTA_M2 lentivirus (VectorBuilder, VB160419-1020mes) and subsequently selected using 1 μg/mL of G418 according to the manufacturer’s protocol (Dharmacon). Clonal populations were generated by limiting dilution under G418 (Corning, 61-8833-100 mg) selection. Clonal populations of OVCAR8 and MDA-MB-231 cells with CMV>tTS/rtTA were again infected with pLV(Tet)-EGFP:T2A:Puro-TRE-hMEF2D lentivirus (VectorBuilder, VB180504-1036gtn). Clonal populations were generated by limiting dilution under puromycin (MilliporeSigma, D-9897-1G) selection. Clones with the best expression efficiency were selected by Western blotting under 1 μg/mL DOX (MilliporeSigma, D-9897-1G) for 48 hours. OVCAR8-MEF2D and MDA-MB-231-MEF2D inducible cells were maintained in RPMI 1640 (Corning, 15-040-CV) supplemented with 10% FBS, G418 (1000 μg/mL for MDA-MB-231 and 500 μg/mL for OVCAR8), and puromycin (2 μg/mL for MDA-MB-231 and 1 μg/mL for OVCAR8).

### RNAi.

ON-TARGETplus pooled siRNAs targeting human HDAC4 (J-003497), HDAC5 (J-003498), and nontargeting control siRNA 2 (D-001810-02) and DharmaFect 4 (T-2004-03) were purchased from GE Dharmacon. First, 70 nM of siRNA and 0.2% DharmaFECT 4 were diluted in Opti-MEM (Thermo Fisher Scientific) individually and then mixed together for 20 minutes at room temperature. Cells were then laid on top of siRNA-DharmaFECT mixture. Cells were lysed to determine target gene expression and prepared for luciferase activity assay 72 hours after transfection.

### IHC staining.

FFPE mouse tissue sections were deparaffinized and rehydrated in gradient ethanol solutions. Antigens were retrieved in Rodent Decloaker (BioCare Medical, RD913M) and microwaved twice in an EZ Retriever System V3 (BioGenex) at 95°C for 5 minutes. Tissues were blocked in PeroxAbolish (BioCare Medical, PXA969M) for 30 minutes, Rodent Block M (BioCare Medical, RBM961L) for 30 minutes, and 5% BSA in PBS for 30 minutes. Tissues were incubated with primary antibody as indicated overnight at 4°C. VisUCyte HRP Polymer IgG (R&D Systems, VC001-025 for mouse, VC003-025 for rabbit) was applied for 30 minutes at room temperature followed by DAB chromogenesis (BioCare Medical, BDB2004L). Tissues were counterstained with CAT hematoxylin (Thermo Fisher Scientific, CATHE-M) for 20 seconds. The slides were then dehydrated through gradient ethanol solutions and 2 passes of xylene and sealed with Permount (Thermo Fisher Scientific, SP15-100).

### Luciferase reporter assay.

MEF2 promoter activity was quantified using a MEF2 reporter assay kit (QIAGEN, 336841 CCS-7024L). Cells were plated, incubated overnight, and then transfected with a mixture of a MEF2-responsive luciferase vector and a constitutively expressing *Renilla* luciferase vector (40:1) for 24 hours. Cells were replated into a 96-well plate, incubated for 16 hours, and then treated with vehicle control, ARN3236 (4 μM), or ARN3261 (4 μM) for different intervals or with different doses of ARN3236 and ARN3261 for 24 hours as indicated. Cells were then lysed for a dual luciferase assay. The relative luciferase activity of MEF2 was calculated by normalizing to *Renilla* luciferase activity. To quantify MEF2 promoter activity with and without knockdown of HDAC4 and HDAC5, cells were transfected with targeting siRNA or control siRNA for 24 hours prior to transfection of a mixture of a MEF2-responsive luciferase and *Renilla* luciferase vectors. Cells were replated into a 96-well plate and then treated with ARN3236 (4 μM) or ARN3261 (4 μM) for 24 hours.

### Immunofluorescence staining.

Cells on 22 × 22 mm coverslips were fixed in 4% formaldehyde in PBS (Thermo Fisher Scientific, J19943-K2) and permeabilized with 0.1% Triton X-100 (MilliporeSigma) in PBS for 15 minutes. Cells were blocked with 5% BSA in PBS for 30 minutes and then stained with antibody overnight at 4°C followed by secondary antibody (Thermo Fisher Scientific, polyclonal, 11034) and DAPI for 1 hour. Coverslips were mounted with Fluoro-Gel with TES buffer (Electron Microscopy Sciences, 50-246-96) and air-dried. HDAC5 nuclear localization was evaluated by measuring nuclear fluorescence intensity of HDAC5. Cells were treated with DMSO, ARN3236 (3 μM), or ARN3261 (5 μM). After 24 hours of incubation, cells were fixed in 4% formaldehyde in PBS. Cells were stained as described above. Images were captured using an Olympus Model IX71 measuring nuclear HDAC4 fluorescence intensity in each cell using ImageJ (NIH). DNA damage visualized by γ-H2AX staining was evaluated by counting nuclear γ-H2AX puncta in each cell. Cells were treated with DMSO, 1 μM of olaparib alone, 4 μM of ARN3261, or 1 μM of ARN3236, or the combination of olaparib and SIK2 inhibitors. After 8 hours of incubation, cells were fixed in 4% formaldehyde in PBS. Cells were stained as described above. Images were captured using an Olympus IX71 microscope and nuclear γ-H2AX puncta in each cell were counted using with Olympus CellSens Dimension software. HDAC5 (catalog 20458) and γ-H2AX (catalog 2577) antibodies were purchased from Cell Signaling Technology.

### Apoptosis.

The percentages of apoptotic cells induced by ARN3236/ARN3261, olaparib, or a combination of both were measured on different ovarian cancer cell lines by FACS using FITC Annexin V/Dead cell Apoptosis kit I (Thermo Fisher Scientific, V13242) according to the manufacturer’s instructions. Briefly, following indicated treatment, cells were harvested and washed once in 1× PBS. Afterward, cells were resuspended in 1× binding buffer containing 5 μL of fluorochrome-conjugated annexin V plus 100 μg/mL propidium iodide. After 15 minutes of incubation at room temperature, cells were centrifuged at room temperature for 3 minutes at 1000*g* and resuspended in 200 μL 1× binding buffer and analyzed with flow cytometry. Stained cells were read on a Gallios analyzer (Beckman Coulter) and 20,000 events were counted.

### Growth of human ovarian and breast cancer xenografts in mice.

Experiments with Hsd Athymic *nu/nu*-*Foxn1^nu^* mice (Envigo stock number 069) were reviewed and approved by the IACUC of MD Anderson Cancer Center.

### SKOv3 and OVCAR8 ovarian cancer xenografts.

A total of 60 female *nu/nu* mice were injected with 5 × 10^6^ SKOv3 cells s.c. or 3.5 × 10^6^ OVCAR8 cells i.p., respectively. After 7 days, mice were randomly assigned to the following treatment groups (*n* = 10): a) vehicle control, b) ARN3236 (40 mg/kg for SKOv3 or 50 mg/kg for OVCAR8 per mouse, 5 times per week), c) ARN3261 (40 mg/kg for SKOv3 or 50 mg/kg for OVCAR8 per mouse, 5 times per week), d) olaparib (50 mg/kg per mouse, 5 times per week), e) ARN3236 combined with olaparib, and f) ARN3261 combined with olaparib. All mice were treated orally with vehicle control, a single agent, or a combination of single agents for 4 weeks (SKOv3 xenograft models) or 6 weeks (OVCAR8 xenograft models) and euthanized with CO_2_ 1 week after completion of treatments. For SKOv3 xenograft models, tumors were measured every week in 2 dimensions using a digital caliper, and the tumor volume was calculated with the following formula: tumor volume (mm^3^) = 0.5 × *ab*^2^ (*a* and *b* being the longest and the shortest diameters of the tumor, respectively). Mice were monitored until tumor burden reached 1500 mm^3^ (ethical endpoint). For OVCAR8 xenograft models, all tumors were weighed immediately after death.

### OC316 ovarian cancer xenografts.

A total of 40 female *nu/nu* mice were i.p. injected with 3.5 × 10^6^ cells. After 7 days of inoculation, tumor-bearing mice were randomly divided into 5 groups (*n* = 10): a) vehicle control, b) ARN3261 (50 mg/kg 5 times per week), c) olaparib (50 mg/kg per mouse, 5 times per week), d) ARN3261 combined with olaparib, and e) ARN3261 combined with olaparib. All mice were treated orally with vehicle control, a single agent, or a combination of single agents for 5 weeks and then continually monitored for survival. Mice were monitored until dyspnea, weight loss, hunched posture, snuffling respiratory sounds, or abdominal breathing were observed (ethical endpoint) for euthanasia.

### MDA-MB-231 breast cancer xenografts.

A total of 40 female *nu/nu* mice were injected with 0.8 × 10^6^ MDA-MB-231 cells into their fourth mammary fat pads. After 7 days, tumor-bearing mice were randomly divided into 5 groups (*n* = 10): a) vehicle control, b) ARN3261 (50 mg/kg 5 times per week), c) olaparib (50 mg/kg per mouse, 5 times per week), d) ARN3261 combined with olaparib, and e) ARN3261 combined with olaparib. All mice were treated orally with vehicle control, a single agent, or a combination of single agents for 6 weeks and then continually monitored for survival. Tumors were measured every week as noted above (SKOv3 xenograft models). Mice were monitored until tumor burden reached 1500 mm^3^ (ethical endpoint).

### HCC-1937 breast cancer xenografts.

A total of 32 female *nu/nu* mice were injected with 4 × 10^6^ cells into their fourth mammary fat pads. After 7 days, tumor-bearing mice were randomly divided into 5 groups (*n* = 8): a) vehicle control, b) ARN3261 (50 mg/kg 5 times per week), c) olaparib (50 mg/kg per mouse, 5 times per week), d) ARN3261 combined with olaparib, and e) ARN3261 combined with olaparib. All mice were treated orally with vehicle control, a single agent, or a combination of single agents for 4 weeks and then continually monitored for survival (ethical endpoint). Tumors were measured every week as noted above (SKOv3 xenograft models). Mice were monitored until tumor burden reached 1500 mm^3^ (ethical endpoint).

### Statistics.

Experiments were repeated 2 or 3 times. Data were plotted using GraphPad Prism 8 and compared using 2-tailed Student’s *t* test and 1-way or 2-way ANOVA. Kaplan-Meier survival analysis of xenograft studies was performed using a log-rank test by GraphPad Prism. Data are presented as mean ± SD unless specified. *P* less than 0.05 was considered significant.

### Study approval.

The mouse xenograft study was reviewed and approved by the IACUC of MD Anderson Cancer Center (IACUC 00001052).

## Author contributions

ZL and RCB designed the study. WM, HY, JMSO, PJR, JM, CGL, XL, KM, EMC, LP, and DSL carried out the experiments. HY, ZL, and RCB analyzed the data, and ZL and RCB wrote the manuscript. HV and AAA provided critical reagents. HC, CI, MC, YT, and YX analyzed bioinformatics data. GAC, HL, AAA, and HV revised the manuscript. All authors approved the manuscript.

## Supplementary Material

Supplemental data

## Version 1 06/01/2022

Electronic publication
