# Supplementary material for: SIK2 inhibition enhances PARP inhibitor activity synergistically in ovarian and triple-negative breast cancers
Source: J Clin Invest. 2022 Jun 1;132(11):e146471. doi: 10.1172/JCI146471 (PMC9151707; doi:10.1172/JCI146471)
Supplement: Supplemental data [file jci-132-146471-s141.pdf]

Supplemental information

SIK2 inhibition synergistically enhances PARP inhibitor activity in ovarian and triple-negative breast cancers

Zhen Lu[1*], Weiqun Mao[1], Hailing Yang[1], Janice M. Santiago-O'Farrill[1], Philip J. Rask[1], Jayanta Mondal[1], Hu Chen[2], Cristina Ivan[1,4], Xiuping Liu[1], Chang-Gong Liu[1], Yuanxin Xi[2], Kenta Masuda[5,6], Eli M Carrami[5,6], Meng Chen[4], Yitao Tang[2,5], Lan Pang [4], David S. Lakomy[1], George A. Calin[4], Han Liang[2,3], Ahmed A. Ahmed[6,7,8], Hariprasad Vankayalapati[9] and Robert C. Bast Jr [1*]

Departments of [1]Experimental Therapeutics, [2]Bioinformatics & Computational Biology, [3]Systems Biology, and [4]Translational Molecular Pathology, University of Texas M.D. Anderson Cancer Center, TX 77030, USA. [5]The University of Texas MD Anderson UTHealth Graduate School of Biomedical Sciences Houston, TX 77030, USA. [6]Ovarian Cancer Cell Laboratory, Weatherall Institute of Molecular Medicine, University of Oxford, Headington, Oxford, OX3 9DS, UK and [7]Nuffield Department of Women's & Reproductive Health, University of Oxford, Women's Centre, John Radcliffe Hospital, Oxford, OX3 9DU, UK and [8]Oxford NIHR Biomedical Research Centre, Oxford, OX4 2PG, UK. [9]Arrien Pharmaceuticals, 9980 South, 300 West, Suite # 200, Salt Lake City, USA.

[*] Corresponding authors.

Address correspondence to Robert C. Bast Jr. and Zhen Lu at Unit 1439, 1400 Pressler Street, Houston, TX 77030; phone: 713-792-7743; FAX: 713-792-7864; E-mail: rbast@mdanderson.org, zlu@mdanderson.org.

Table S1. Source and culture medium of cell lines

Cell Line	Source	Culture Medium	Tissue Classification
OC316	Gordon Mills	RPMI1640 10%PBS	Ovarian Cancer
OVCAR5	Gordon Mills	RPMI 1640 10%FBS	Ovarian Cancer
MDA-2774	Gordon Mills	RPMI 1640 10%PBS	Ovarian Cancer
OVCAR8	Gordon Mills	RPMI 1640 10%PBS	Ovarian Cancer
2008	Gordon Mills	RPMI 1640 10%PBS	Ovarian Cancer
OVCAR3	ATCC	RPMI 1640 10%PBS	Ovarian Cancer
A2780	ATCC	RPMI 1640 10%FBS	Ovarian Cancer
ES2	ATCC	RPMI1640 10%PBS	Ovarian Cancer
IGROV1	ATCC	RPMI 1640 10%FBS	Ovarian Cancer
Caov3	ATCC	DMEM 10%PBS	Ovarian Cancer
OV90	ATCC	105 + 199 10%PBS	Ovarian Cancer
TOV21G	ATCC	105 + 199 10%PBS	Ovarian Cancer
TOV112D	ATCC	105 + 199 10%PBS	Ovarian Cancer
OAW42	ATCC	105 + 199 10%PBS	Ovarian Cancer
OAW28	ATCC	DMEM 10%PBS	Ovarian Cancer
SKOv3	ATCC	RPMI 1640 10%FBS	Ovarian Cancer
DOV13	Robert Bast	DMEM 10%PBS	Ovarian Cancer
HCC5030	Adi F. Gazdar	RPMI 1640 10%FBS	Ovarian Cancer
HCC5032	Adi F. Gazdar	RPMI 1640 10%FBS	Ovarian Cancer
HCC5044	Adi F. Gazdar	RPMI 1640 10%FBS	Ovarian Cancer
HCC5075	Adi F. Gazdar	RPMI 1640 10%FBS	Ovarian Cancer
HCC1937	ATCC	RPMI 1640 10%FBS	Breast Cancer
MDA-MB-231	ATCC	RPMI 1640 10%FBS	Breast Cancer
BT549	ATCC	RPMI 1640 10%FBS	Breast Cancer
MDA-MB-468	ATCC	RPMI 1640 10%FBS	Breast Cancer
MDA-MB-436	ATCC	RPMI 1640 10%FBS	Breast Cancer
Cal-51	Creative Bioarray	DMEM 10%FBS	Breast Cancer
SUM149PT [1]	BioIVT	Ham's F-12 5%FBS	Breast Cancer
SUM1315MO2 [2]	BioIVT	Ham's F-12 5%FBS	Breast Cancer
NOE72 [3]	Robert Bast	105 + 199 10%FBS	Normal Ovarian
NOE119L [3]	Robert Bast	105 + 199 10%FBS	Normal Ovarian
HMEC16620 [4]	Robert Bast	HuMEC 10%FBS	Normal Breast
SKOv3 SIK2 KO	Ahmed Ahmed	RPMI 1640 10%FBS	Ovarian Cancer
SKOv3 SIK2 KO CTRL	Ahmed Ahmed	RPMI 1640 10%FBS	Ovarian Cancer
OVCAR8 SIK2 KO	Ahmed Ahmed	RPMI 1640 10%FBS	Ovarian Cancer
OVCAR8 SIK2 KO CTRL	Ahmed Ahmed	RPMI 1640 10%FBS	Ovarian Cancer
SKOv3 SIK2 OE	Ahmed Ahmed	RPMI 1640 10%FBS	Ovarian Cancer
SKOv3 SIK2 OE CTRL	Ahmed Ahmed	RPMI 1640 10%FBS	Ovarian Cancer
OVCAR8 SIK OE	Robert Bast	RPMI 1640 10%FBS	Ovarian Cancer
OVCAR8 SIK2 OE CTRL	Robert Bast	RPMI 1640 10%FBS	Ovarian Cancer
DT40 PARP-1 -/- [5]	Shunichi Takeda	DMEM 10%PBS	Avian cells
DT40 PARP-1 -/- +PARP1 [5]	Shunichi Takeda	DMEM 10%PBS	Avian cells

1. 10 mM HEPES, 5 μg/mL insulin and 1 μg/mL hydrocortisone
2. 10 mM HEPES, 10 ng/mL EGF and 5 μg/mL insulin
3. 20 ng/mL EGF
4. HuMEC basal serum free medium (Gibco, #12753-018) and HuMEC supplement kit (Gibco, #12753-013)
5. 0.05 mM 2-mercaptoethanol

Table S2. The IC50s of inhibitors and concentration ratio of SIK2 inhibitors to olaparib

Cell line	ARN3236 (µM) (IC50)	ARN3261 (µM) (IC50)	Olaparib (µM) (IC50)	ARN3236 : Olaparib	ARN3261 : Olaparib
**OVCAR8**	1.396	1.765	6.396	1:0.5	1:0.5
**SKOv3**	1.231	2.843	55.51	1:12.5	1:12.5
**HCC5032**	2.243	2.397	14.52	1:3	1:1
**OC316**	1.259	2.25	4.91	1:5	1:2
**IGROV1**	1.084	1.605	2.83	1:5	1:2
**MDA-2774**	1.081	0.592	6.289	1:5	1:2
**OV90**	3.869	5.428	8.364	1:1	1:2
**DOV13**	2.811	1.995	32.16	1:6	1:3
**OVCAR3**	2.828	2.29	3.788	1:1	1:1
**OAW28**	1.714	3.400	3.658	1:1	1:1.3
**MDA-MD-231**	2.369	1.182	4.474	1:0.5	1:0.5
**BT549**	2.02	2.1	13.7	1:4	1:4
**Cal-51**	-	2.68	2.7	-	1:2
**MDA-MB-468**	-	5.329	1.588	-	1:1
**MDA-MB-436**	-	3.665	0.098	-	1:0.25
**SUM149PT**	-	34.76	106.1	-	1:20
**SUM1315MO2**	-	2.746	0.845	-	1:3
**HCC1937**	-	10.03	31.05	-	1:10
**NOE72**	5.745	4.161	6.383	1:0.5	1:0.75
**NOE119L**	4.805	5.860	15.06	1:0.5	1:0.75
**HMEC16620**	3.6	5.3	1.2	1:0.5	1:0.25

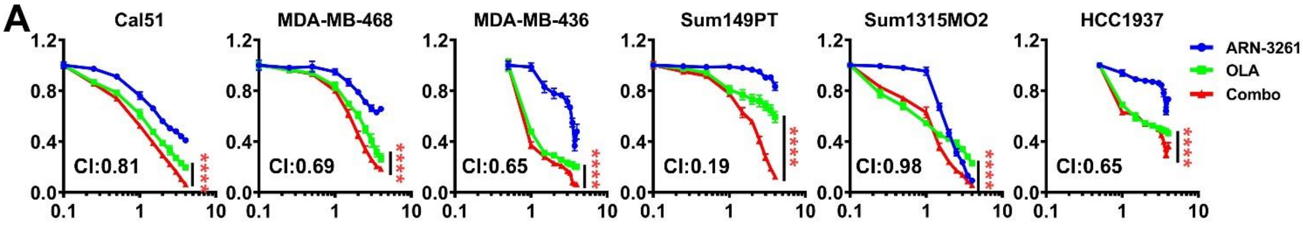

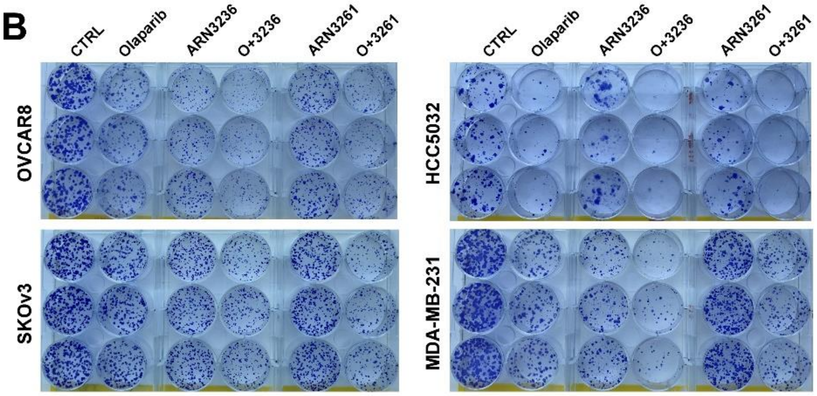

Clonogenic assay

Cell lines	Duration (Day)	Inhibitor (uM) X time		
		3236	3261	Olaparib
SKOv3	10-12	0.5X1	3x2	3X1
OVCAR8	10-12	0.5X1	2x2	0.75X1
HCC5032	10-12	0.75X1	3x2	0.625X1
MDA-MB-213	20-22	0.75X1	4x2	0.75X1

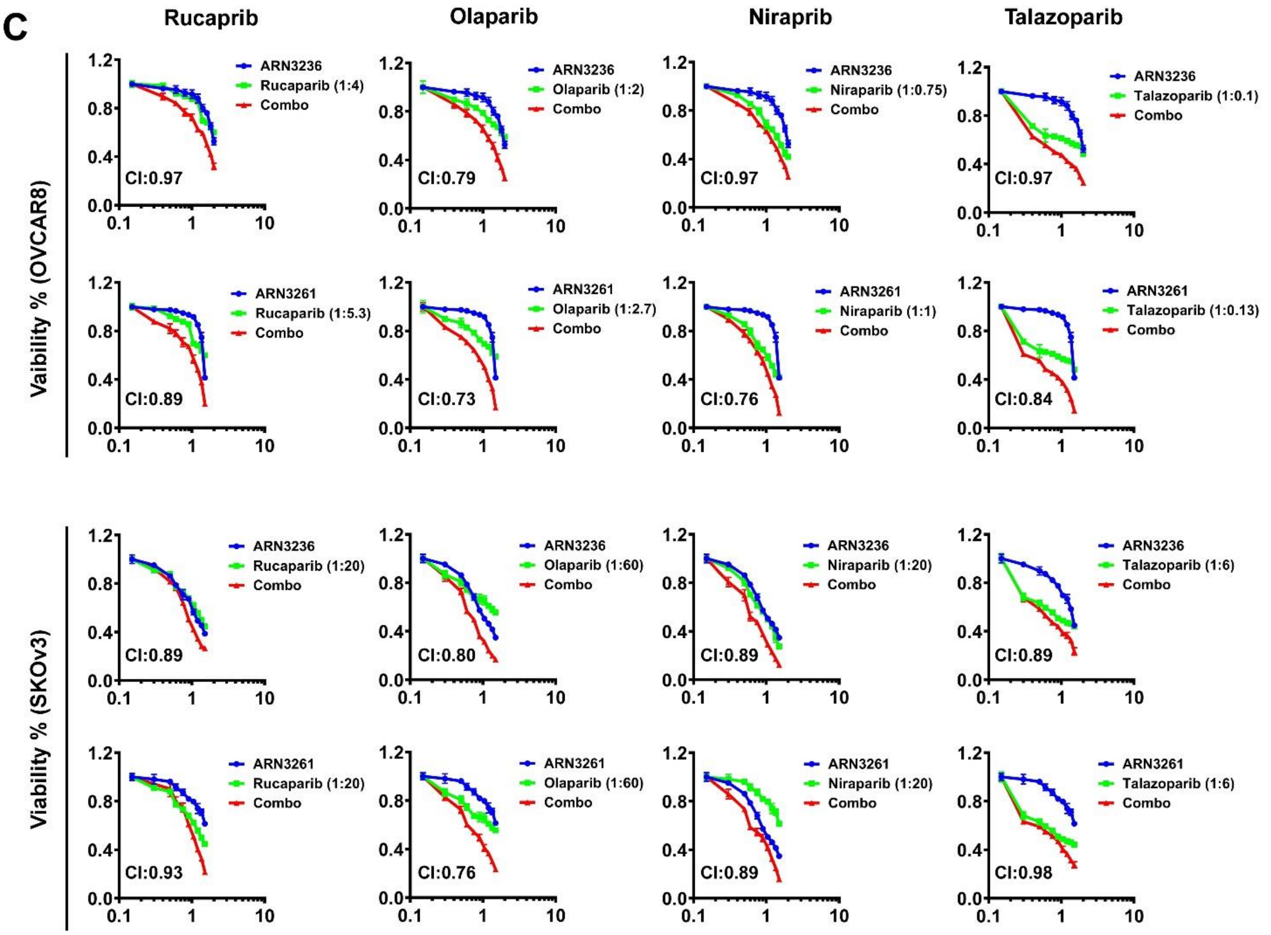

Figure S1. SIK2 inhibitors enhance rucaparib, olaparib, niraparib and talazoparib sensitivity in ovarian cancer. (**A**) Dose-response curves for ARN3261 (blue), olaparib (green) or ARN3261 combined with olaparib (red) for 96 hrs in 6 breast cancer cell lines. The IC50s of inhibitors and concentration ratios of SIK2 inhibitors to olaparib used for each cell line are listed in table S2. The statistical significance between olaparib alone and SIK2 inhibitor combined with olaparib was calculated with two-way ANOVA Tukey's multiple comparisons. **** $p<0.0001$. Red stars indicate SIK2 inhibitor + olaparb enhancing the effect of olaparib alone; A combination index (CI) at ED 90 was calculated using CalcuSyn software. Representative data are from one experiment with four replicates per treatment. Experiments were repeated three times with similar results. (**B**) Representative images of clonogenic assay in four cancer cell lines are presented. SKOv3, OVCAR8, HCC5032 and MDA-MB-231 cells were treated with olaparib, ARN3236, ARN3261 alone, or olaparib plus ARN3236 or ARN3261 at the concentrations indicated for 10-22 days. (**C**) Dose-response curves of ARN3236/ARN3261 (blue), PARP inhibitors (rucaparib, olaparib, niraparib or talazoparib) (green) or ARN-3236/ ARN3261 combined with PARP inhibitor (red) for 96 hrs in OVCAR8 and SKOv3 ovarian cancer cells. A combination index (CI) was calculated using CalcuSyn software at ED 90. Representative data were from one experiment with four replicates per experiment. Experiments were repeated twice.

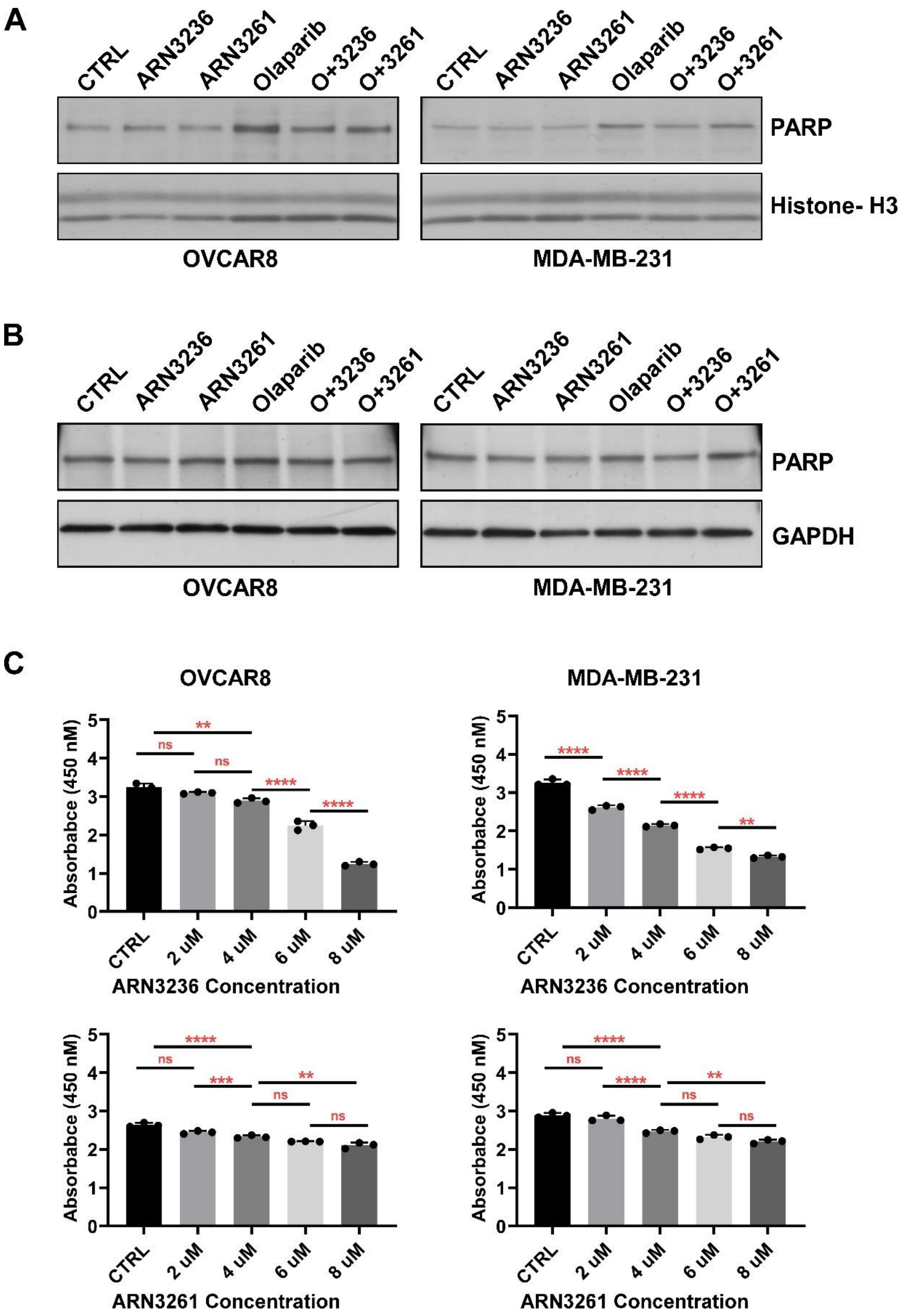
A
CTRL
ARN3236
ARN3261
Olaparib
O+3236
O+3261
PARP
Histone- H3
OVCAR8
MDA-MB-231
B
PARP
GAPDH
C
OVCAR8
MDA-MB-231
Absorbabce (450 nM)
CTRL
2 uM
4 uM
6 uM
8 uM
ARN3236 Concentration
ARN3261 Concentration
ns
**
***
****

Figure S2. Effect of ARN326, ARN3261 and olaparib on PARP1 enzyme activity and trapping. (A) PARP1-trapping in OVCAR8 and MDA-MB-231 cells. Cells were treated with ARN3236, ARN3261, olaparib alone or olaparib+ARN3236 or ARN3261 for 72 hrs. The concentrations of ARN3236, ARN3261 and olaparib were 4 µM, 4 µM, and 6 µM, respectively. Western blot analysis of chromatin-bound fractions of PARP1. Experiments were repeated twice with similar results. (**B**) Western blot analysis of PARP1 protein expression. (**C**) The dose-response effect of olaparib and SIK2 inhibitor on PARP1 enzyme activity. OVCAR8 and MDA-MB-231 cells were treated with SIK2 inhibitors for 26 hrs as indicated. The columns indicate the mean of activity and the bars indicate the S.D. The statistical significance was calculated with Tukey's one-way ANOVA multiple comparisons (^{ns} p>0.05, ** p<0.01, *** p<0.001, **** p<0.0001). Representative experiments were from one experiment with three replicates. Experiments were repeated three times with similar results.

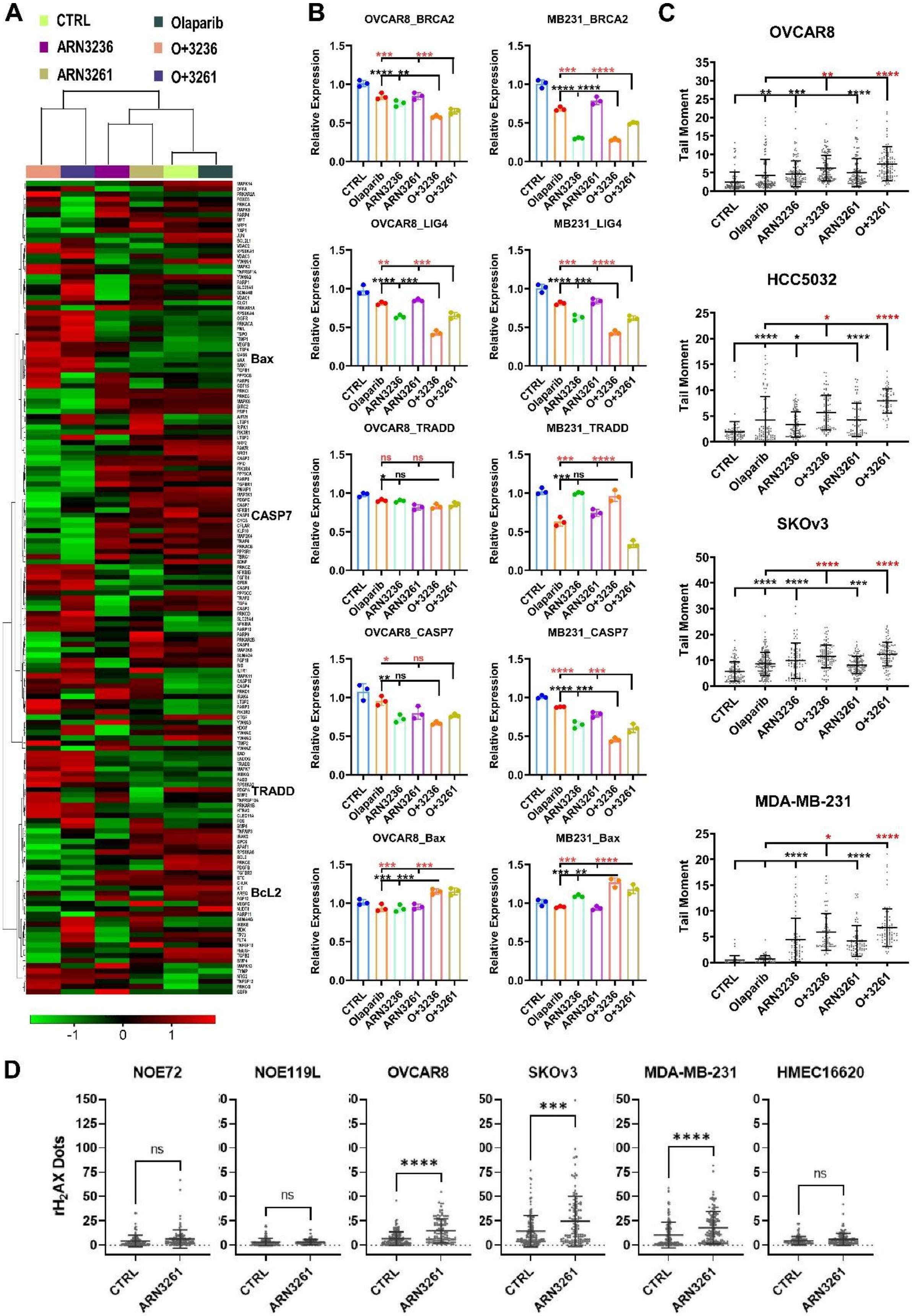

A
CTRL
ARN3236
ARN3261
Olaparib
O+3236
O+3261
Bax
CASP7
TRADD
BcL2
-1
0
1
B
OVCAR8_BRCA2
MB231_BRCA2
OVCAR8_LIG4
MB231_LIG4
OVCAR8_TRADD
MB231_TRADD
OVCAR8_CASP7
MB231_CASP7
OVCAR8_Bax
MB231_Bax
Relative Expression
CTRL
Olaparib
ARN3236
ARN3261
O+3236
O+3261
C
OVCAR8
HCC5032
SKOv3
MDA-MB-231
Tail Moment
D
NOE72
NOE119L
OVCAR8
SKOv3
MDA-MB-231
HMEC16620
rH2AX Dots
ns
****
***
CTRL
ARN3261

Figure S3. ARN3236 and ARN3261 enhances olaparib-induced DNA double-strain breaks and apoptosis. (**A**) A heatmap of unsupervised hierarchical clustering of differently expressed genes associated with apoptosis. The map contains changes that are color coded with red corresponding to up-regulation and green to down-regulation. (**B**) Analysis of DNA repair and apoptosis genes. Gene expression was analyzed using RT-qPCR in SKOv3 and OVCAR8 ovarian cancer cells. Cells were treated with ARN3236, ARN3261, olaparib alone or olaparib+ARN3236 or ARN3261 for 72 hrs. The concentrations of ARN3236, ARN3261 and olaparib are 4μM (2 times), 4μM (3 times), and 15μM (2 times), respectively. The columns indicate the mean of RNA expression and the bars indicate the S.D. One-way ANOVA Tukey's multiple comparisons were performed (* $p<0.05$, ** $p<0.01$, *** $p<0.001$, **** $p<0.0001$). Representative experiments were from one independent experiment with 3 technical repeats per treatment. Experiments were repeated three times with similar results. (**C**) Quantification of DNA damage using comet assays. Cells were plated and treated with ARN3236, ARN3261 or olaparib on the comet slides for a total of 48 hrs with and without olaparib for 16 hrs before harvest. HCC5032, OVCAR8 and SKOv3 were incubated with 1 μM of ARN3236 and MDA-MB-231 cells with 0.5 μM. All 4 cell lines tested were treated with 4 μM of ARN3261 and 5 μM of olaparib. Olive Tail Moment was measured using CaspLab1.2.3β2 software. The columns indicate the mean of tail moments and the bars indicate the S.D. One-way ANOVA Tukey's multiple comparisons were performed (* $p<0.05$, ** $p<0.01$, *** $p<0.001$, **** $p<0.0001$). Representative data were from three independent experiments with a total of 100-200 cells per treatment. (**D**) Quantification of DNA damage (γ-H2AX). Endogenous γ-H2AX was stained with anti-γ-H2AX antibody in the cells treated with single agent or combined for 24 hrs. The concentration of ARN3261 is 1 μM. γ-H2AX dots were quantified with OLYMPUS CellSens Dimension software. T-test (two-tails) was performed between treatment and control (ns p>0.05, *** p<0.001, **** p<0.0001). Experiments were from two independent experiments with a total of 100 cells per treatment.

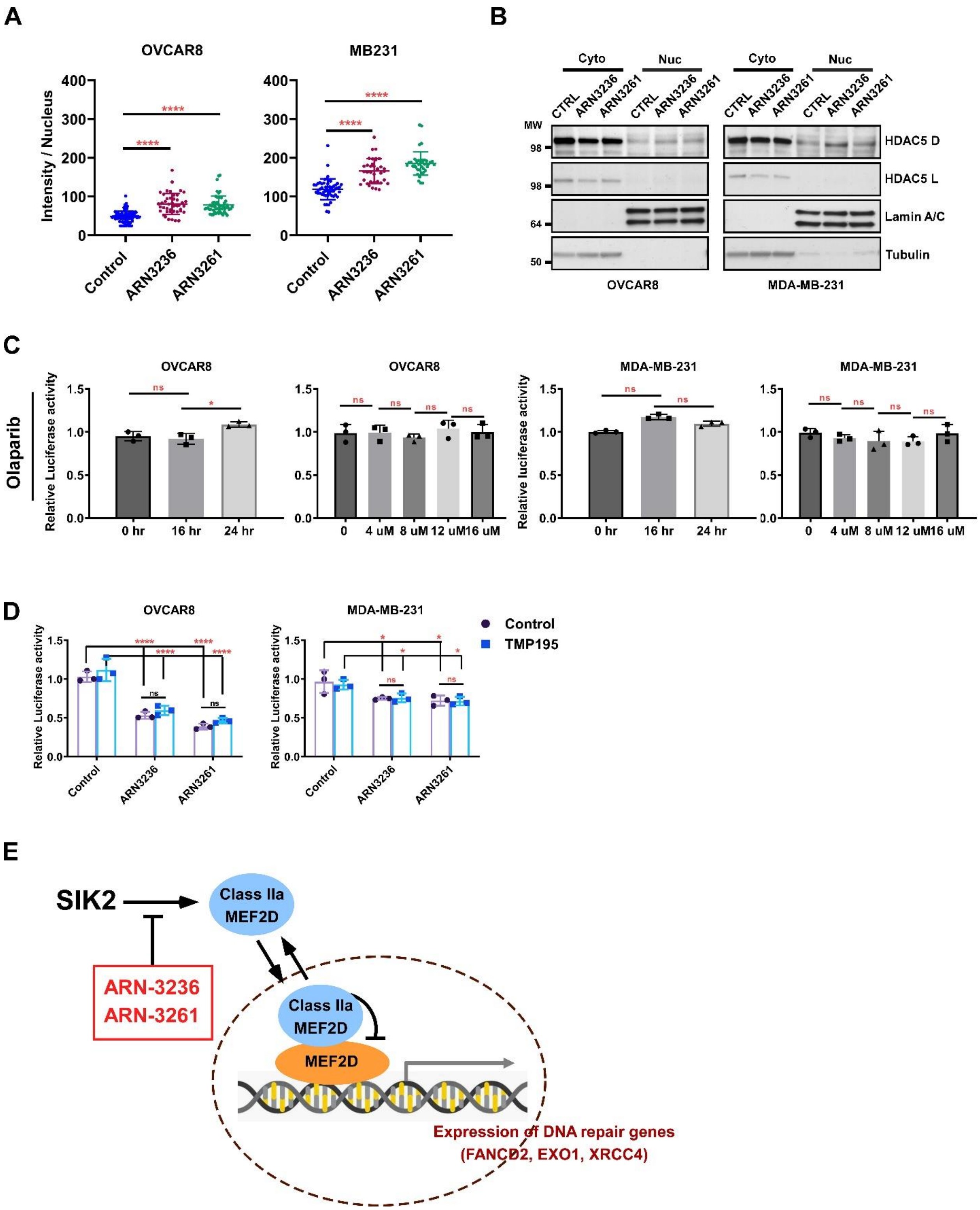
A
OVCAR8
MB231
Intensity / Nucleus
Control
ARN3236
ARN3261
****
B
Cyto
Nuc
CTRL
ARN3236
ARN3261
MW
98
64
50
HDAC5 D
HDAC5 L
Lamin A/C
Tubulin
OVCAR8
MDA-MB-231
C
Olaparib
OVCAR8
MDA-MB-231
Relative Luciferase activity
Relative luciferase activity
ns
*
0 hr
16 hr
24 hr
0
4 uM
8 uM
12 uM
16 uM
D
OVCAR8
MDA-MB-231
Control
TMP195
Relative Luciferase activity
****
ns
*
Control
ARN3236
ARN3261
E
SIK2
Class IIa
MEF2D
ARN-3236
ARN-3261
MEF2D
Expression of DNA repair genes
(FANCD2, EXO1, XRCC4)

Figure S4. ARN3236 and ARN3261 decrease phosphorylation of HDAC4/5/7 and promoter activity of MEF2 transcription factors. (**A**) Detection of HDAC5 localization with and without SIK2 inhibitors using immunofluorescence staining. Nuclear fluorescence intensity was measured by ImageJ (related to Figure 7A). Data are from three independent experiments with a total of 100-150 cells per group. The middle solid lines indicate the mean of fluorescent intensity. The top and bottom solid lines indicate the S.D. One-way ANOVA Tukey's multiple comparisons analysis was performed (*** $p<0.001$, **** $p<0.0001$). (**B**) Detection of HDAC5 localization with and without SIK2 inhibitors using cell fractionation. OVCAR8 and MDA-MB-231 cells were treated with ARN3236 (6 µM) or ARN3261 (5 µM) for 26 hrs. Total cell lyses were collected for cell fractionation and cytoplasmic extracts and nuclear extracts were subjected to western analysis using the antibodies indicated. (D and L indicate dark and light exposure, respectively). Experiments were repeated twice with similar outcomes. (**C**) Quantification of MEF2 promoter activity (related to Figure 7B). Cells were plated and after overnight incubation, then transfected with a mixture of a MEF2-responsive luciferase construct and a constitutively expressing *Renilla* luciferase construct (40:1) (QIAGEN) for 24 hrs. Cells were re-plated into 96 well plates and then treated with olaparib (4 µM) for different time intervals or with different doses of olaparib for 24 hrs as indicated. Cells were lysed for dual luciferase assay. The relative luciferase activity of MEF2 was calculated by normalizing to *Renilla* luciferase activity. The columns indicate the mean of MEF2 luciferase activity, and the bars indicate the S.D. One-way ANOVA Tukey's multiple comparisons analysis was performed (^{ns} $p>0.05$, * $p<0.05$). Representative experiments were from one independent experiment with 3 technical repeats per treatment. Experiments were repeated twice with similar outcomes. (**D**) Quantification of MEF2 promoter activity (related to Figure 7C). Cells were treated with TMP195 for 24 hrs prior to transfection of a mixture of a MEF2-responsive luciferase construct and *Renilla* luciferase construct. Cells were re-plated into 96 well plate and then treated with ARN3236 (4 µM) and ARN3261 (4 µM) for 24 hrs. Measurement of luciferase activity was performed, quantified and analyzed as described in (C). The bars indicate the S.D. Two-way ANOVA Dunnett's multiple comparisons analysis was performed (^{ns} $p>0.05$, * $p<0.05$, **** $p<0.0001$). Representative data were from one experiment with 3 replicates per treatment. Experiments were repeated twice with similar outcomes. (**E**) Working model. SIK2 inhibitor inhibits class IIa HDAC / MEF2D-mediated downregulation of genes that are associated with DNA repair.

A

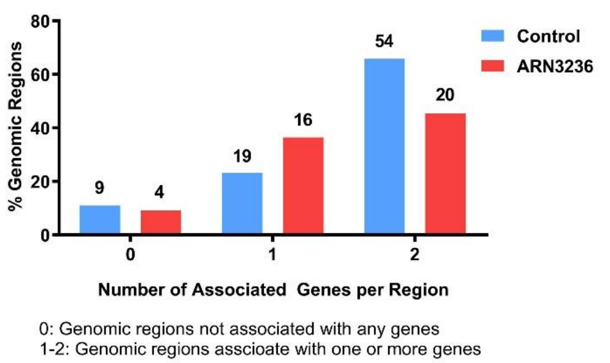

B

GO terms

MEF2D ARN-3236	P-value
Positive regualtion of cell differentiation (PRKCH, H2AFY2, AHSP, GATA1)	0.0023
Negative regulation of cell apoptotic process (PRKCH, RIPK2, CIB1, IL6ST, GATA1, MT3))	0.0124
V(D)J recombination (RAG2)	0.0311
Positive regulation of DNA repair (FANCD2)	0.035
**MEF2D Control**	
Regulation of tumor necrosis factor mediated signaling pathway (CYLD, SYK)	0.0055
DNA damage induced protien phosphorylation (MAP2K6)	0.0178
Positive regulation of cell apoptotic process (USP17L24, CYLD)	0.0326

C

D

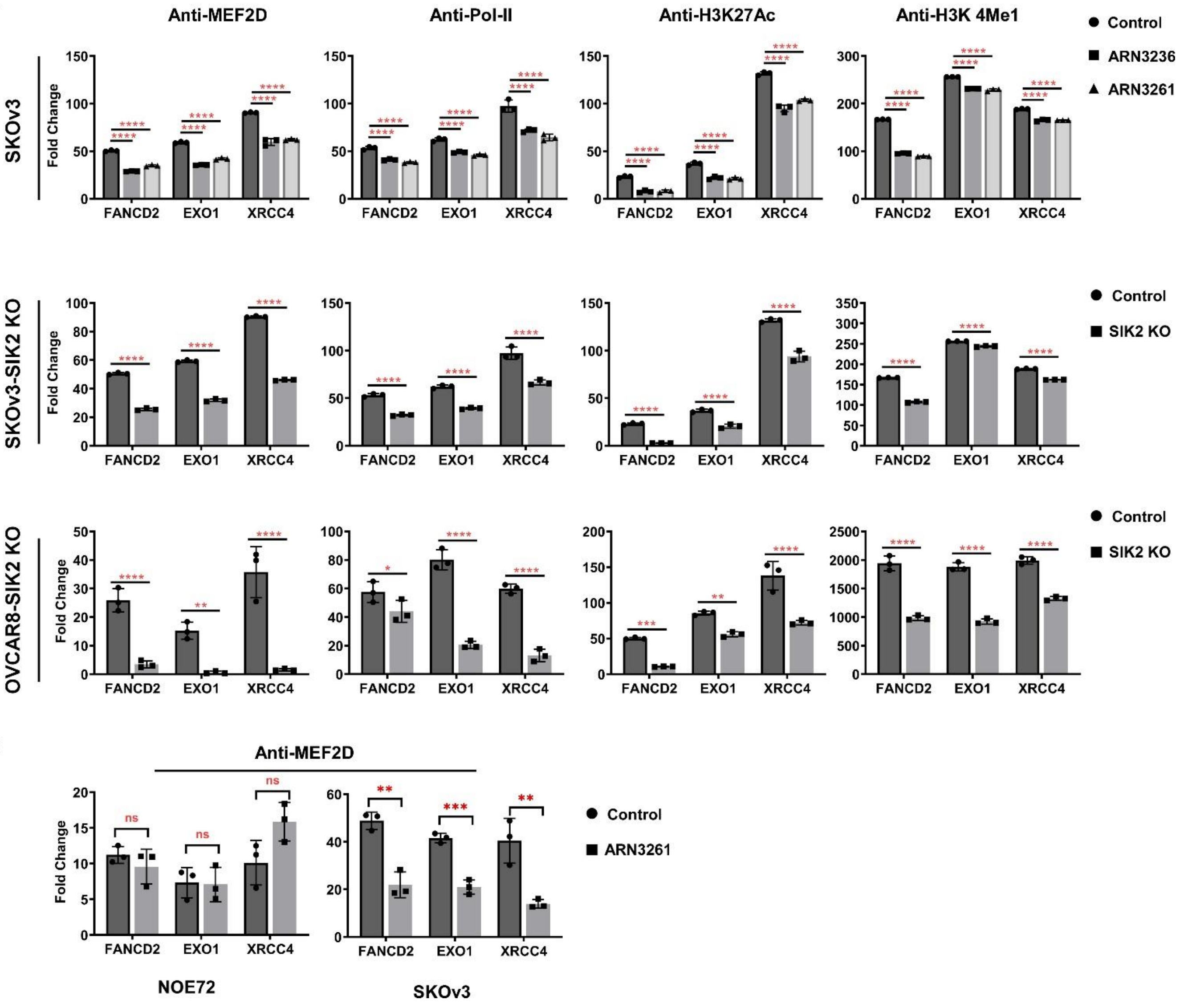

Figure S5. **SIK2 inhibition alters MEF2D transcription factor-mediated downstream signaling** (related to Figure 8). (**A**) MEF2D-binding sites in human ovarian cancer cells. (**B**) GO analysis of MEF2D-bound genes. (**C**) Chip analysis of FANCD2, EXO1 and XRCC4 genes in SKOv3, SKOv3- and OVCA8- SIK2 knockout cells. SKOv3, SKOv3- and OVCA8- SIK2 knockout cells were treated with and without ARN3236 (6 µM) or ARN3261 (4 µM) for 48-50 hrs and then harvested subjecting to Chip with normal IgG, MEF2D, Pol-II, H3K27Ac or H3KMe1 antibody. Chip pull-downed samples were analyzed by RT-qPCR as indicated. The columns indicate the mean of relative fold changes (Fold change=2-DDCt, Chip signal relative to the IgG background signal) and the bars indicate the S.D. Two-way ANOVA Dunnett's multiple comparisons were performed (* $p<0.05$, ** $p<0.01$, *** $p<0.001$, **** $p<0.0001$). Representative experiments were from one independent experiment with 3 replicates per treatment. Experiments were repeated twice. (**D**) Chip analysis of FANCD2, EXO1 and XRCC4 genes in NOE72 and SKOv3 cells. NOE72 and SKOv3 cells were treated with and without ARN3236 (6 µM) or ARN3261 (4 µM) for 48-50 hrs and then harvested subjecting to Chip with normal IgG and MEF2D. Chip pull-downed samples were analyzed by RT-qPCR as indicated. The columns indicate the mean of relative fold changes as described in (C). The bars indicate the S.D. Two-way ANOVA Dunnett's multiple comparisons were performed (^{ns} $p>0.05$, ** $p<0.01$, *** $p<0.001$).

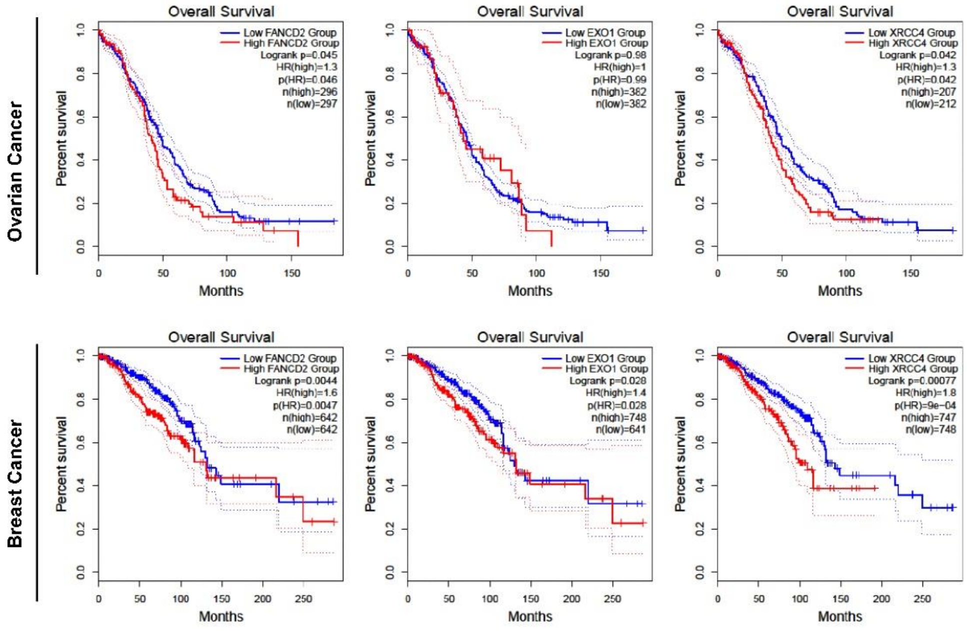

Figure S6. **Clinical data analysis by log-rank test (http://gepia.cancer-pku.cn/).** Kaplan Meier survival curves of FANCD2, EXO1 and XRCC1 in ovarian and breast cancer.

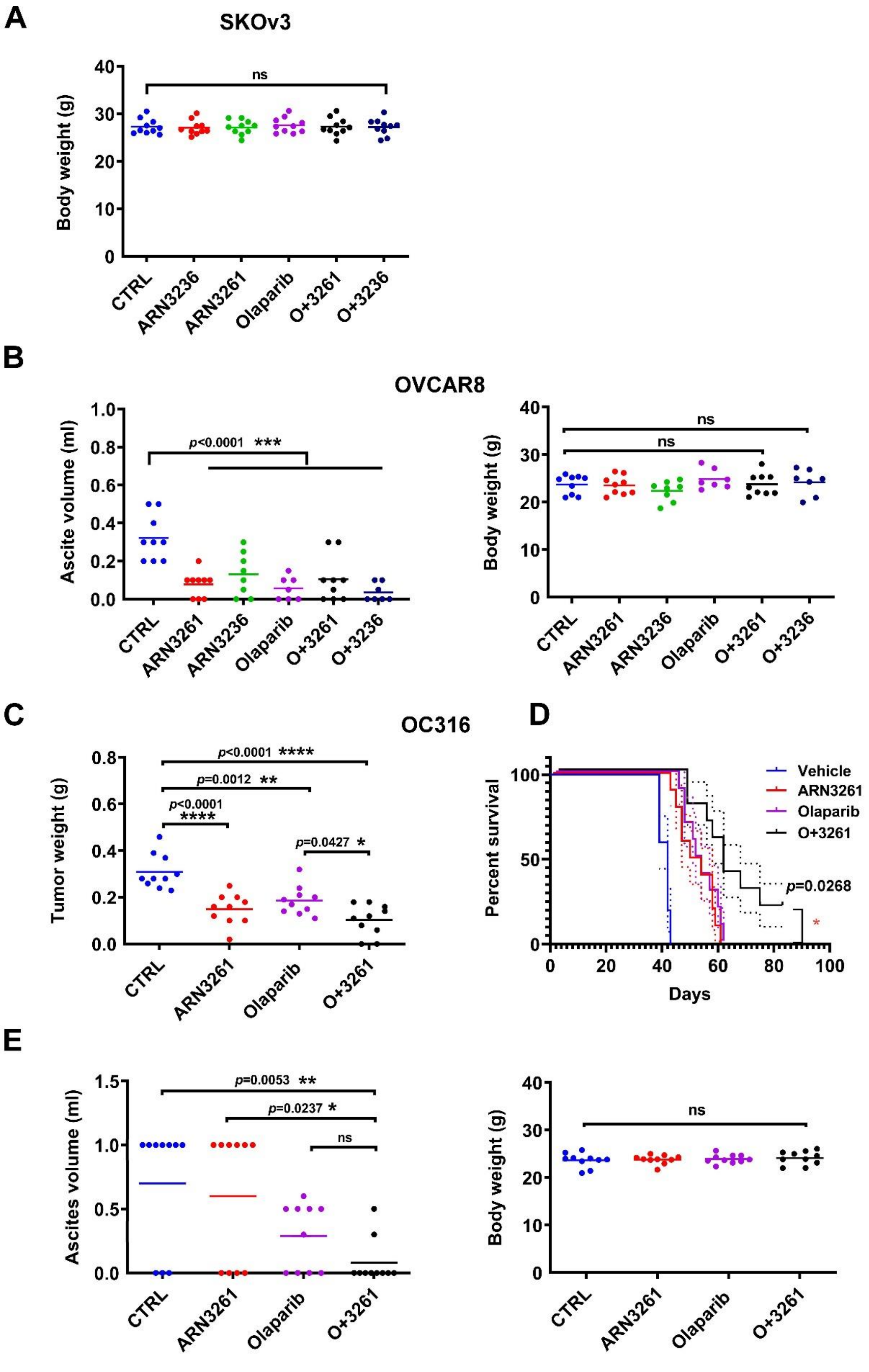

A
SKOv3
Body weight (g)
ns
CTRL
ARN3236
ARN3261
Olaparib
O+3261
O+3236
B
OVCAR8
Ascite volume (ml)
p<0.0001 ***
CTRL
ARN3261
ARN3236
Olaparib
O+3261
O+3236
Body weight (g)
ns
ns
C
OC316
Tumor weight (g)
p<0.0001 ****
p=0.0012 **
p<0.0001 ****
p=0.0427 *
CTRL
ARN3261
Olaparib
O+3261
D
Percent survival
Vehicle
ARN3261
Olaparib
O+3261
p=0.0268
*
Days
E
Ascites volume (ml)
p=0.0053 **
p=0.0237 *
ns
CTRL
ARN3261
Olaparib
O+3261
Body weight (g)
ns

Figure S7. Co-administration of SIK2 inhibitor and olaparib is synergistic in vivo (related to Figure 10). (**A**) Mouse body weights and ascites volume for SKOv3 xenografts. The mouse body weights were measured at the end of experiments. The middle solid lines indicate the mean of body weight. One-way ANOVA Tukey's multiple comparison analysis was performed (ns p>0.05). (**B**) Mouse body weights and ascites volume for OVCAR8 xenografts. The mouse body weights, and ascites volume were measured at the end of experiments. The middle solid lines indicate the mean of ascites volume (left) or body weight (right). One-way ANOVA Tukey's multiple comparison analysis was performed (^{ns} $p>0.05$, *** $p<0.001$). (**C**-**D**) Tumor growth of OC316 cells and survival of tumor bearing mice. Tumor-bearing mice were randomized into 4 treatment groups after 7-days of tumor growth. Mice (n=20/treatment, 10 for tumor volume and 10 for survival) were treated with ARN3236, ARN3261, olaparib or a combination as indicated for 5 weeks. Mice bearing OC316 xenografts were sacrificed and tumors were weighed after completing 5 weeks of treatment. Tumor weight after different treatments was plotted as mean ± S.D. (*p<0.05; ** p<0.01). One-way ANOVA Tukey's multiple comparison analysis was performed for tumor growth (C). Survival curves were generated by GraphPad Prism 6. (ns p>0.05, *p<0.05; ** p<0.01). A log-rank test was performed for survival curve comparison (D). (**E**) Mouse body weight and ascites volume for OC316 model. The mouse body weights, and ascites volume were measured at the end of experiments. The middle solid lines indicate the mean of ascites volume (left) or body weight (right). One-way ANOVA Tukey's multiple comparison analysis was performed (ns p>0.05, *p<0.1, ** p<0.01).

Materials and Methods

Alkaline Single-Cell Agarose Gel Electrophoresis (Comet) Assays. 1-2x10^5 cells in 6-well plates were treated with DMSO, SIK2 inhibitors (ARN3236 or ARN3261), olaparib or the combination of SIK2 inhibitor and olaparib. Treatment conditions were as follows: 1 µM of ARN3236 for HCC5032, OVCAR8 and SKOv3 and 0.5 µM of ARN3236 for MDA-MB-231 for 48 hrs; 5 uM of ARN3261 for all four cell lines for 48 hrs; and 5 µM of olaparib for all four cell lines for 16 hrs before harvest. Cells were trypsinized and resuspended at 2x10^5/mL in cold PBS without Ca^{2+} and Mg^{2+}. Cells were mixed with pre-warmed comet agarose at a 1:10 (v/v) ratio. 10 uL of cell agarose mixture was plated onto comet slides pre-coated with 75 uL of agarose and chilled at 4ºC for 15 min to set. Cells were lysed in 25 mL of Lysis buffer at 4ºC for 2 hrs and washed with alkaline solution (pH 10). Comet slides were electrophoresed in cold alkaline solution at 20V for 15 min. Slides were rinsed with water and dried in 70% ethanol for 5 min. Slides were then stained with Vista Green DNA dye and viewed using an Olympus epifluorescence microscope with a FITC filter. Images were captured using a 20x objective. A 3-Well OxiSelect™ Comet Assay kit was purchased from Cell Biolabs, Inc (STA-351). Experiments were run in triplicate and Olive Tail Moment was measured using CaspLab1.2.3β2 software (CaspLab.com). Olive Tail Moment = Tail DNA% x Tail Length. 50-200 Cells were measured for each treatment and experiments were repeated twice independently to ensure reproducibility.

ChIP and RT-qPCR analysis. OVCAR8, MDA-MB-231, SKOv3, OVCAR8-SIK2 KO or SKOv3-SIK2 KO cells (2 million) were cultured on a150 cm plate and treated the next day either with vehicle control or with ARN3236 (4 µM) or ARN3261(5 µM) for 48 hrs. ChIP assays were performed using the Magna ChIP A Kit (Millipore, 17-610). Briefly, cells after treatment with ARN3236 or ARN3261 for 48 hrs were incubated with 1% formaldehyde for 10 min at room temperature and neutralized with 1× glycine. Nuclei were isolated and sonicated to obtain 200 - 1000 bp DNA fragments using the QSONICA sonicator for 30 cycles with 10 seconds pulses at 100% amplitude with 2 min of incubation on ice between pulses. For individual ChIP assays, 100 µg of soluble chromatin per sample was immunoprecipitated with 8 µg of mouse IgG control antibody (Santa Cruz, sc-2025), 8 µg of rabbit control antibody (Millipore, PP64B), 8 µg of MEF2D antibody (Santa Cruz, sc-27115 3X), 8 µg of RNA polymerase II antibody (Abcam, ab817), 6 µg of Histone H3 (acetyl K27) antibody (Abcam, ab45173) or 6 µg of Histone H3 (tri methyl K4) (Abcam, ab8580). For ChIP-Sequence, 500 µg of chromatin per sample was immunoprecipitated with 40 µg of MEF2D antibody. Input determined from 1% of the cell lysate was used as a negative control.

ChIP-Sequence and Analysis. Sequencing was performed by the Sequencing and Microarray Facility (SMF) at MD Anderson Cancer Center. Briefly, Indexed libraries were prepared from 20 ng of Diagenode Biorupter sheared ChIP DNA using the KAPA Hyper Library Preparation Kit (Kapa Biosystems, Inc). Libraries were amplified by 8 cycles of PCR and then size distribution was assessed using the 4200 TapeStation High Sensitivity D1000 ScreenTape (Agilent Technologies) and quantified using the Qubit dsDNA HS Assay Kit (ThermoFisher). The indexed libraries were multiplexed, 10 libraries per pool. The pool was quantified by

qPCR using the KAPA Library Quantification Kit (KAPA Biosystems) then sequenced on the Illumina NextSeq500 sequencer using the High-output 75 single read configuration. The raw reads were first preprocessed to remove sequencing adapters and low-quality reads. The trimmed reads were then mapped to human reference genome hg19 using bowtie (1), with only uniquely mapped reads retained. The ChIP-seq occupancy profiles were generated by MACS 1.4 (2) with the "--wig" parameter, and were normalized to 20 million total reads. Duplicated reads were automatically removed by MACS. ChIPseq peaks were called by MACS with p-value set to 1e-8. Peaks were annotated to associated genes according to their relative locations. T associated genes were identified as ChIP-seq target genes. Further functional analysis on these genes were carried out, including gene ontology (GO) analysis using DAVID (3, 4). The enriched DNA binding motifs in ChIP-seq peak regions were identified and compared with known motifs using HOMER v4.8 (5).

mRNA-Sequence and Analysis. Poly(A) containing mRNA sequencing was performed by the Sequencing and ncRNA Shared Resource at the MD Anderson Cancer Center. The indexed mRNA sequencing libraries were prepared from total RNA with RIN > 9.0 using Illumina TrueSeq stranded mRNA library preparation kits (Illumina, RS-122-2101 and RS-122-2102), following guidance of an Illumina Truseq stranded mRNA protocol. In Brief, 200 ng of total RNA were used for poly(A) mRNA enrichment using oligo(dT) coated magnetic beads. The enriched and purified mRNA was fragmented into small pieces using divalent cations at elevated temperature. The cleaved RNA fragments were then reverse transcribed into first strand cDNA by reverse transcriptase using random hexamer primers for RT priming and reverse transcription, followed by second strand cDNA synthesis using DNA polymerase I and RNase H. These double strand cDNA fragments were end-repaired and then adenylated at 3' ends with the addition of a single 'A' base to prevent self-ligation during subsequent ligation to the Illumina index-specific adapters that has a single "T" at 3' end which provides complementary overhang for ligating the adapter to the fragment. The raw library products were purified and enriched by PCR to create the final cDNA sequencing library. The indexed individual sequencing library was quantified using an Agilent Bioanalyzer Highly Sensitive DNA assay. To ensure the sufficient data coverage for high, medium and low copy transcripts, twelve indexed mRNA libraries were pooled and sequenced on an Illumina Nextseq 500 sequencer using TruSeq High Output Kit V2 150 cycles (FC-404-2001) in Paired-end E75 sequencing configuration. The raw data bcl files were de-multiplexed and converted into fastq files using Illumina bcl2fastq2 conversion V 2.19 software (illumina). We used FastQC to perform a quality control of the FASTQ files and STAR (GRCh38, Gencode25 and STAR 2.6.1b) to map the reads against the reference genome and count the number of reads uniquely mapping to each gene, for each sample. Heatmap plots of selected genes showing their variation among different samples were generated in R, version 3.5.1, using the heatmap 2 function of g plots library. Public domain of gene pathways (https://www.qiagen.com/us/) was used to retrieve genes related to apoptosis and DNA Damage repair. Gene Ontology Enrichment Analysis for differentially expressed genes was performed using the web-based tool Enrichr (6, 7).

References

1. Langmead B, Trapnell C, Pop M, and Salzberg SL. Ultrafast and memory-efficient alignment of short DNA sequences to the human genome. *Genome Biol.* 2009;10(3):R25.
2. Liu T. Use model-based Analysis of ChIP-Seq (MACS) to analyze short reads generated by sequencing protein-DNA interactions in embryonic stem cells. *Methods Mol Biol.* 2014;1150:81-95.
3. Wang J, Huang D, Zhou Y, Yao H, Liu H, Zhai S, et al. CAUSALdb: a database for disease/trait causal variants identified using summary statistics of genome-wide association studies. *Nucleic Acids Res.* 2020;48(D1):D807-D16.
4. Huang da W, Sherman BT, and Lempicki RA. Systematic and integrative analysis of large gene lists using DAVID bioinformatics resources. *Nat Protoc.* 2009;4(1):44-57.
5. Heinz S, Benner C, Spann N, Bertolino E, Lin YC, Laslo P, et al. Simple combinations of lineage-determining transcription factors prime cis-regulatory elements required for macrophage and B cell identities. *Mol Cell.* 2010;38(4):576-89.
6. Chen EY, Tan CM, Kou Y, Duan Q, Wang Z, Meirelles GV, et al. Enrichr: interactive and collaborative HTML5 gene list enrichment analysis tool. *BMC Bioinformatics.* 2013;14:128.
7. Kuleshov MV, Jones MR, Rouillard AD, Fernandez NF, Duan Q, Wang Z, et al. Enrichr: a comprehensive gene set enrichment analysis web server 2016 update. *Nucleic Acids Res.* 2016;44(W1):W90-7.
